# Fis suppresses late-stage virulence gene expression in *Yersinia pseudotuberculosis* at environmental temperatures

**DOI:** 10.1371/journal.ppat.1014105

**Published:** 2026-03-25

**Authors:** Soheila Javadi, Stephan Pienkoß, Dominik Meggers, Andrea Wimbert, Vivian B. Brandenburg, Pascal Dietze, Sina Schäkermann, Lilo Greune, Petra Dersch, Franz Narberhaus

**Affiliations:** 1 Microbial Biology, Ruhr University Bochum, Bochum, Germany; 2 Bioinformatics Group, Ruhr University Bochum, Bochum, Germany; 3 Applied Microbiology, Ruhr University Bochum, Bochum, Germany; 4 Institute of Infectiology, Center for Molecular Biology of Inflammation (ZMBE), University of Münster, Münster, Germany; INSERM U1220, FRANCE

## Abstract

The nucleoid-associated protein Fis is a key transcriptional regulator in Gram-negative bacteria that supports rapid adaptation to environmental changes. In *Yersinia pseudotuberculosis*, Fis plays a critical, yet poorly understood role in virulence. Here, we present a comparative transcriptomic analysis of *Y. pseudotuberculosis* wild type and *fis* deletion mutant at environmental (25°C) and host-relevant (37°C) temperatures. Our data show that Fis modulates the expression of more than 600 genes across 16 functional categories. Notably, Fis exerts reciprocal, temperature-dependent control over virulence genes, including those encoding the type III secretion system (T3SS) and *Yersinia* effector proteins (Yops), flagella biosynthesis, and cell adherence/invasion factors. Functional assays revealed that *fis* deletion disrupts this regulatory balance, producing a host-defense-like state at 25°C characterized by complete loss of motility, upregulation of the virulence master regulator LcrF, aberrant Yop secretion, impaired phagocytosis by host cells, and increased pathogenicity in the *Galleria mellonella* infection model. These findings establish Fis as a central regulator that coordinates motility and early host-cell engagement while preventing premature activation of antiphagocytic defenses, thereby optimizing the initial stages of infection.

## Author summary

Bacterial pathogens must precisely control virulence gene expression to transition successfully between environmental reservoirs and host infection. In *Yersinia pseudotuberculosis*, the nucleoid-associated protein Fis plays a crucial role in coordinating this balance. Our study reveals that Fis functions as a temperature-sensitive regulatory switch. At moderate environmental temperatures, it represses virulence genes involved in immune evasion while promoting flagella biosynthesis and cell adhesion. Deletion of *fis* disrupts this regulation, inducing an inappropriate virulent state at 25°C, in which bacteria lose motility, prematurely secrete virulence factors, evade phagocytosis, and exhibit increased pathogenicity. These findings identify Fis as a key regulator that maintains *Yersinia* in a non-virulent, environmentally adapted state outside the host, ensuring precise spatiotemporal control over motility and virulence to optimize both environmental survival and infection potential.

## Introduction

Pathogenic bacteria rely on complex regulatory networks to fine-tune gene expression in response to the diverse environmental conditions encountered during both free-living and host-associated lifestyles. A crucial aspect of this regulation is the control of gene expression by *trans*-acting regulatory elements, such as transcription factors [1]. Transcription factors can be classified as local or global regulators, depending on the number of their target genes and their mode of action [2–4]. Local transcription factors are typically encoded in close genomic proximity to their regulated target genes and bind with high affinity to specific regulatory sequences. In contrast, global regulators interact with a broader range of binding sites, often with lower specificity [1,3]. Among global regulators, nucleoid-associated proteins (NAPs) play a critical role in gene regulation through their diverse DNA-binding activity. By bending, wrapping, or bridging DNA, NAPs can influence transcription [5,6].

One prominent NAP in Gram-negative bacteria is Fis (factor for inversion stimulation), initially identified for its role in stimulating DNA inversion and phage lambda recombination [7]. Beyond this, Fis is responsible for the organization and maintenance of the nucleoid structure [8, 9]. Its expression peaks during the early exponential growth phase and declines in the stationary phase, reflecting its function as a growth-phase-dependent transcriptional regulator [8,10,11]. Fis is a small, homodimeric DNA-binding protein that influences transcription both directly and indirectly. It binds to promoter regions to either promote or inhibit RNA polymerase recruitment and also modulates DNA supercoiling, affecting gene expression on a broader scale [5,9,12]. Genome-wide studies in *Escherichia coli* and *Salmonella enterica* serovar Typhimurium have identified more than 1,000 Fis-binding sites, located either upstream or within coding regions [13–16]. These studies highlight Fis as a global regulator influencing a wide range of cellular processes, including energy metabolism, motility, chemotaxis, and stress responses [15,17–19].

In addition to its role in general gene regulation, Fis is also involved in bacterial virulence. In *Salmonella*, Fis positively regulates genes encoding the type III secretion system (T3SS), a critical apparatus for host-cell invasion and immune evasion [16,20,21]. A similar function has been observed in *Pseudomonas aeruginosa*, where Fis promotes the expression of *exsA* coding for the master virulence regulator under infection-relevant conditions [22]. Furthermore, in *Yersinia pseudotuberculosis,* deletion of *fis* resulted in reduced resistance to reactive oxygen species (ROS) and impaired colonization of the murine spleen and liver [23]. Despite these findings, the precise role of Fis in *Yersinia* species remains poorly understood.

The *Yersinia* genus comprises three human pathogenic species: *Y. pestis*, the causative agent of plague, and the enteric species *Y. pseudotuberculosis* and *Y. enterocolitica*, which are responsible for gastrointestinal infections [24,25]. These species rely on highly sophisticated regulatory networks to precisely modulate gene expression in response to environmental signals such as temperature, pH, and nutrient availability [26–28]. Among these cues, temperature is particularly crucial for enteric *Yersinia* species, as it signals the transition from an external environment to a warm-blooded host, triggering the expression of virulence factors. For example, *Y. pseudotuberculosis* undergoes extensive transcriptional reprogramming in response to temperature shifts or upon entering host tissues, affecting the expression of more than 300 genes [29,30].

At moderate temperatures below 30°C, enteric *Yersinia* species express early-stage virulence factors to produce the primary internalization factor invasin (Inv) and the attachment-invasion locus (Ail) proteins [31,32]. These factors facilitate infection initiation by promoting bacterial adherence to and penetration of the intestinal epithelial barrier [32,33]. In contrast, late-stage virulence factors remain repressed at low temperatures, primarily due to the thermosensitive modulator YmoA and the histone-like nucleoid-structuring protein H-NS, which inhibit expression of the gene coding for the main virulence regulator LcrF [34–37].

Upon host entry, the temperature shift to 37°C and other host-associated cues activate the production of multiple regulatory factors essential for intracellular survival and efficient dissemination in deeper tissues [29,30,38]. Among these, the thermally regulated virulence factor LcrF, controlled by an RNA thermometer, activates the transcription of T3SS genes encoded on the _~_70 kb *Yersinia* virulence plasmid pYV (also named as pIB1) [39–41]. The T3SS, known as the injectisome, enables the direct injection of *Yersinia* outer proteins (Yops) into host immune cells such as phagocytes, allowing the bacteria to evade immune responses [42,43]. These Yop effector proteins counteract the host´s innate immune defenses by disrupting signal transduction pathways involved in programmed cell death, disorganizing the host cell cytoskeleton, and blocking inflammatory cytokine production [44–46].

In this study, we identified the NAP Fis as a key regulatory factor in *Y. pseudotuberculosis,* ensuring distinct temperature-dependent expression profiles between environmental (25°C) and host (37°C) states. Whole transcriptome RNA sequencing of the wild type (wt) strain and a *fis* deletion mutant at 25 and 37°C revealed that Fis functions as both a positive and negative global regulator in *Yersinia*, similar to its role in other Gram-negative enteric bacteria. Notably, we provide the first evidence that Fis precisely represses the expression of the *ysc/yop* regulon under environmental conditions. Physiological and virulence assays further demonstrated that *fis* deletion abolishes this repression, leading to a host immune defense state even at moderate temperatures (25°C). Additionally, our data indicate that Fis positively regulates *Yersinia* motility and cell adhesion/invasion factors. This highlights a temperature-dependent reciprocal regulation of motility/initial cell contact and immune defense strategies, with Fis acting as a central transcriptional switch within the *Yersinia* regulatory network.

## Results

### *fis* expression is temperature- and growth phase-dependent

**Fig 1:**
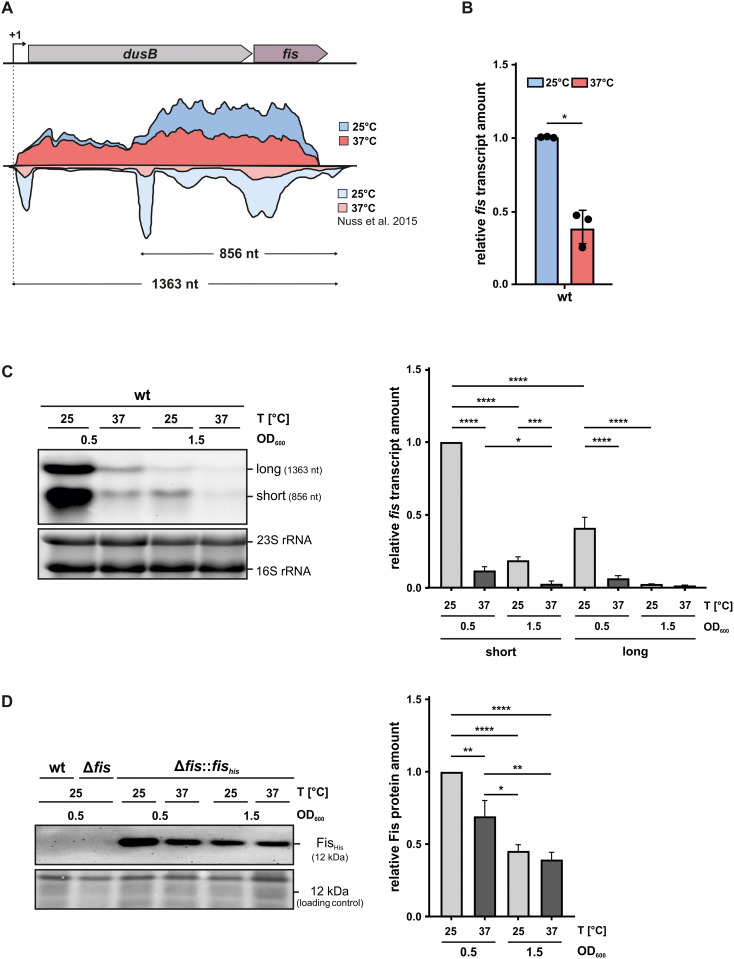
*fis* expression decreases at 37°C and during stationary growth phase. **(A)** Schematic representation of the bicistronic *dusB-fis* operon with transcript profiles derived from RNA-seq data from this study and [29] visualized by the Artemis genome browser. Two transcripts are represented: a full-length 1363 nt long *dusB-fis* transcript and a shorter 856 nt *fis*-specific transcript. The profiles display RNA abundance at 25°C (blue) and 37°C (red). **(B)** Comparison of *fis* transcript levels during early exponential growth phase at 25 and 37°C. Samples were taken at an OD_600_ of 0.5 in *Y. pseudotuberculosis* wt grown in LB medium, followed by RNA isolation and qRT-PCR analysis. Transcript levels were normalized to *fis* transcript levels at 25°C and to the reference genes *nuoB* and *hrpA.* Data represent the mean and standard deviation (SD) of three independent biological replicates. Statistical significance was tested using *t*-test (*p < 0.01) **(C)** *fis* transcript levels in the wt were determined by Northern blot analysis during early (OD_600_ 0.5) and late (OD_600_ 1.5) growth phases at 25 and 37°C. Equal RNA amounts were loaded, and a *fis*-specific probe was used for detection. GelRed-stained ribosomal RNAs (23S and 16S rRNAs) served as loading controls. Transcript intensities were quantified by Bio-Rad software (right panel) from three independent experiments and normalized to the short *fis* transcript level at 25°C and OD_600_ 0.5. Statistical significance differences were determined using the one-way ANOVA test (****p < 0.0001) **(D)** Fis protein levels in a chromosomally integrated His-tagged *fis*-complemented *fis* mutant Δ*fis*::*fis_his_* were analyzed by Western blot under the same conditions as in (C). Ponceau S-stained membrane served as loading control. Fis protein amounts were quantified using Bio-Rad software (right panel) from three independent biological replicates and normalized to Fis levels at 25°C and OD_600_ 0.5. Significant differences were determined using the one-way ANOVA test (****p < 0.0001).

To investigate the regulatory role of Fis in *Y. pseudotuberculosis*, we first examined its expression profile under different conditions. The *fis* gene is part of the bicistronic *dusB*-*fis* operon on the chromosome (Fig 1A). Previous RNA-seq analyses at single-nucleotide resolution identified a transcription start site approximately 32 nucleotides (nt) upstream of *dusB*, indicating that a single promoter regulates both genes, as observed in other members of the *Enterobacteriaceae* family [10,11,29]. Despite this shared promoter, two distinct transcripts have been detected: a full-length _~_ 1400 nt transcript spanning both genes and a shorter *fis*-specific transcript with _~_ 860 nt [10,11]. Consistent with these findings, our RNA-seq data at 25 and 37°C confirmed the presence of both transcripts in *Y. pseudotuberculosis*. Remarkably, while both were expressed at comparable levels at 37°C, the short transcript was more abundant at 25°C (Fig 1A). qRT-PCR and Northern blot analyses using a *fis*-specific probe validated these observations, confirming the presence of two transcripts and revealing higher *fis* transcript levels at 25°C compared to 37°C (Fig 1B-C and S1 Fig). Additionally, transcript levels significantly declined from early (OD_600_ 0.5) to late exponential growth (OD_600_ 1.5) (Fig 1C), which aligns with reports from other Gram-negative bacteria [10,21,47,48].

Western blot analysis detecting the product of a chromosomally integrated *fis-*His-tag fusion in a complemented *fis* mutant (Δ*fis::fis*_*his*_) further demonstrated higher Fis production at 25°C during early exponential growth (OD_600_ 0.5), which decreased at 37°C and during late exponential phase (OD_600_ 1.5) (Fig 1D).

### Fis controls global gene expression in *Yersinia*

Fluctuations in environmental conditions, such as temperature and nutrient availability, are prevalent during transitions between external niches and the host, and drive *Yersinia* to undergo extensive transcriptional reprogramming to ensure adaptability and pathogenicity [29]. In bacteria, transcriptional modifications are orchestrated by the cooperation of multiple regulators, including NAPs. Given Fis’ role as a key regulator in various Gram-negative bacteria, particularly in virulence, we sought to characterize its function in *Yersinia*. To determine whether Fis functions as a global regulator in *Yersinia*, we constructed a *fis* deletion mutant (Δ*fis*) and performed comparative RNA-seq analyses with the wt strain under environmental (25°C) and host (37°C) conditions during early (OD_600_ 0.5) and late (OD_600_ 1.5) exponential growth phases.

**Fig 2:**
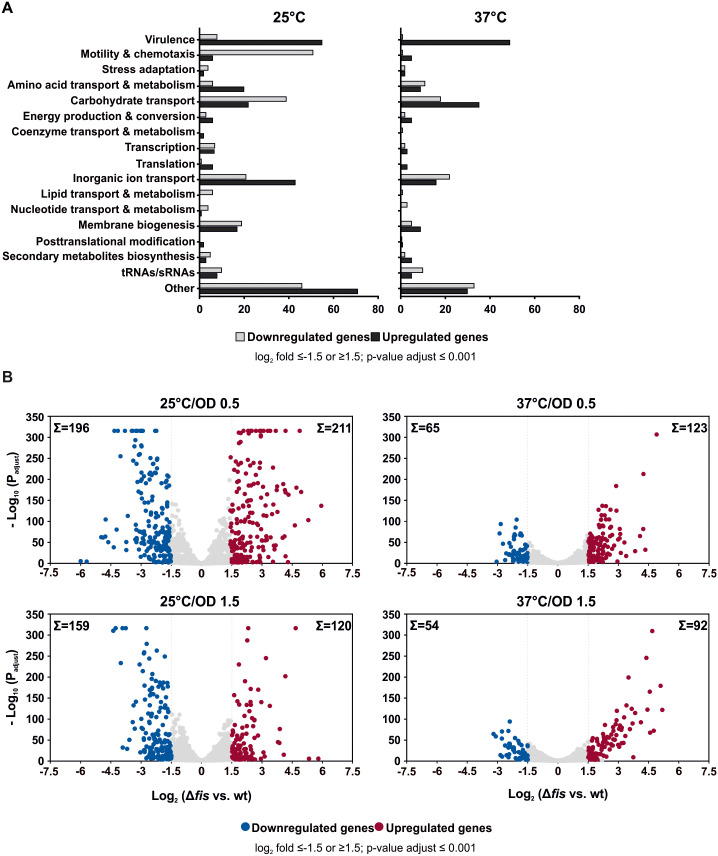
Fis controls global gene expression in response to environmental changes. (A) KEGG pathway classification of differentially expressed genes in the *Y. pseudotuberculosis fis* mutant compared to the wt strain at 25°C (left panel) and 37°C (right panel). Gene categories are indicated on the left. **(B)** Volcano plots illustrate down- and upregulated genes in the *fis* mutant under indicated conditions. The x-axis represents the log_2_(∆*fis*/wt) fold change in gene expression and the y-axis shows the log_10_(adjusted p-value) for statistical significance. Non-significant genes are depicted in gray. Blue and red dots indicate down- and upregulated genes, respectively. RNA-seq was performed on three biological replicates.

Our RNA-seq identified 613 genes that were differentially expressed between the wt and *fis* mutant strains (log_2_ fold change ≤ -1.5 or ≥1.5, adjusted p-value ≤ 0.001). Functional clustering using the Kyoto Encyclopedia of Genes and Genomes (KEGG) database grouped these genes into 16 functional categories, highlighting Fis as a key global regulator involved in diverse cellular processes, including motility, virulence, stress response, and multiple metabolic pathways (Fig 2A). A nearly equal distribution of upregulated or downregulated genes suggests that Fis acts as both an activator and a repressor. Notably, its regulatory impact was strongest at 25°C during early exponential growth, affecting 407 genes (Fig 2B and S2 Fig). This observation is congruent with the increased *fis* expression under this condition (Fig 1 and S1 Fig).

**Fig 3:**
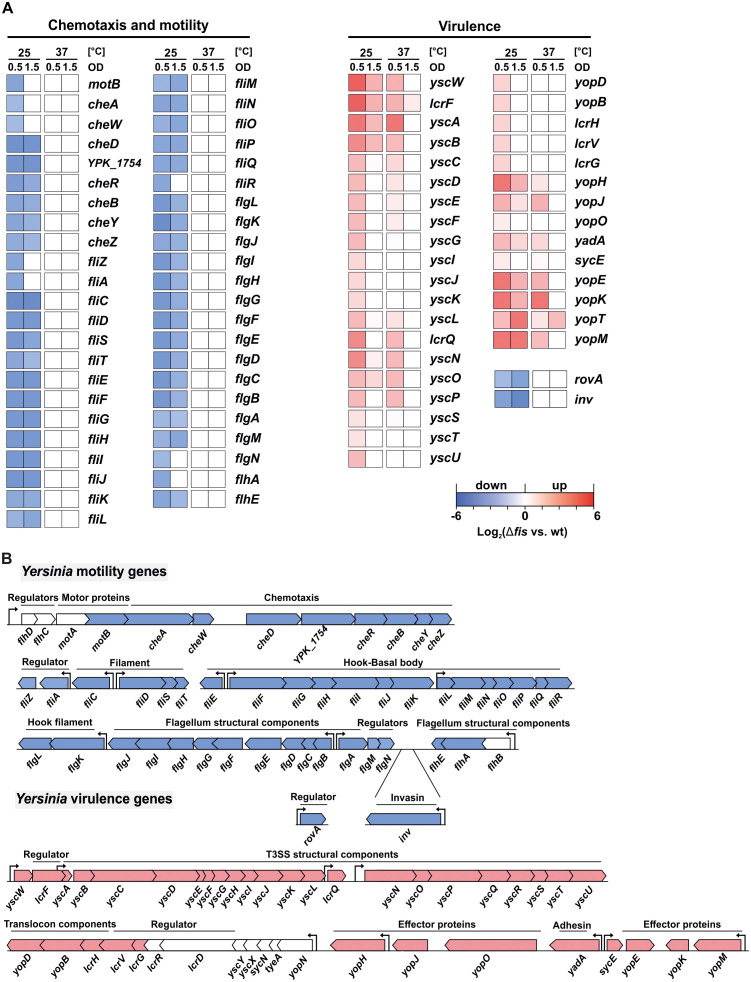
Temperature-dependent inverse expression profile of motility and virulence genes in the absence of Fis. (A) Heatmap highlighting the differentially expressed genes in the *fis* mutant compared to the wt strain involved in chemotaxis, motility, and virulence. Transcription levels were determined during the early (OD_600_ 0.5) and late exponential (OD_600_ 1.5) growth phases at 25 and 37°C. Expression was calculated relative to the wt grown under the same conditions and indicated as log_2_ values. Different colors indicate different fold changes in gene expression. Blue boxes represent downregulated expression, whereas red boxes highlight upregulation. White boxes indicate non-significant changes. **(B)** The lower section depicts the operon organization of flagellar and virulence genes in *Y. pseudotuberculosis* YPIII, with a schematic color scheme representing overall expression change trends in the *fis* mutant. Arrows indicate the transcription start sites of each operon [29].

Intriguingly, the most affected genes in the *fis* mutant were involved in chemotaxis and motility (63 genes), and virulence (113 genes) (Fig 2A). In *Yersinia*, flagellum biosynthesis follows a hierarchical regulatory cascade involving over 60 chromosomally encoded genes, categorized into class I, II, and III (S3A Fig). These classes include genes for key regulators, structural components, and the chemosensory machinery [43,49,50]. Our RNA-seq data revealed that class II genes, encoding the basal body, hook structural proteins, and the regulators FliA and FlgM, as well as class III genes encoding chemotaxis machinery, filament, and motor-force generators, were significantly downregulated in the *fis* mutant at 25°C (Fig 3A-B). Although the differential expression of class I genes, including the regulator operon *flhDC* (*YPK_1745, YPK_1746*), did not reach the defined significance threshold (log_2_ fold change ≤ -1.5), their expression followed the general downregulation pattern observed for other motility-associated genes (S1 Dataset). The RNA-seq results were further validated by qRT-PCR, which confirmed decreased transcript levels of motility-related genes in the *fis* mutant compared to the wt and complementation strain (S3B Fig). Notably, *invA*, a gene co-regulated with flagellar genes (Fig 3B) and encoding the virulence-associated invasin protein A, also exhibited reduced expression (Fig 3A) [51,52]. Consistently, *rovA*, coding for the positive regulator of *invA*, was also downregulated (Fig 3A) [31,35].

Surprisingly, the absence of Fis led to a substantial upregulation of virulence plasmid (pYV)-encoded genes, including those coding for the T3SS machinery, effector proteins (Yops), and the adhesin protein YadA, particularly at 25°C (Fig 3A). These genes are typically induced at 37°C and upon host uptake and cell contact [53,54], suggesting that Fis functions as a repressor of the T3SS regulon under moderate temperatures in the wt. Notably, among the upregulated genes, the *yscW*-*lcrF* operon (Fig 3B), encoding the master virulence regulator LcrF, exhibited a pronounced, almost 4-fold increase in transcript levels in the *fis* mutant at 25°C and a 2.6-fold increase at 37°C (Fig 3A). In addition, 19 other pYV-encoded genes, including those coding for hypothetical proteins, transposases, and pseudogenes, were also upregulated (S2 Dataset).

Overall, these transcriptomic data suggest that the absence of Fis disrupts *Yersinia*´s global expression profile, leading to a premature virulent expression state even at 25°C.

Previous studies have reported that the gene dosage of virulence plasmid-encoded genes can increase through amplification of the virulence plasmid copy number in response to elevated temperatures and under virulence-inducing conditions [55,56]. We therefore examined whether deletion of *fis* affects the copy number of the virulence plasmid, which could account for the observed upregulation of virulence genes, particularly at 25°C. To address this, we quantified the plasmid copy number using quantitative PCR [55]. As expected, the copy number increased to 3–4 copies at 37°C in both the wt and *fis* mutant strains. However, similar to the wt, no increase in plasmid copy number was observed in the *fis* mutant at 25°C (S4 Fig). These results indicate that the transcriptional upregulation of virulence genes in the *fis* mutant is not due to changes in plasmid copy number but instead reflects a Fis-dependent regulatory mechanism that represses expression of plasmid-encoded genes under non-induced conditions.

### Fis promotes motility in *Yersinia*

**Fig 4:**
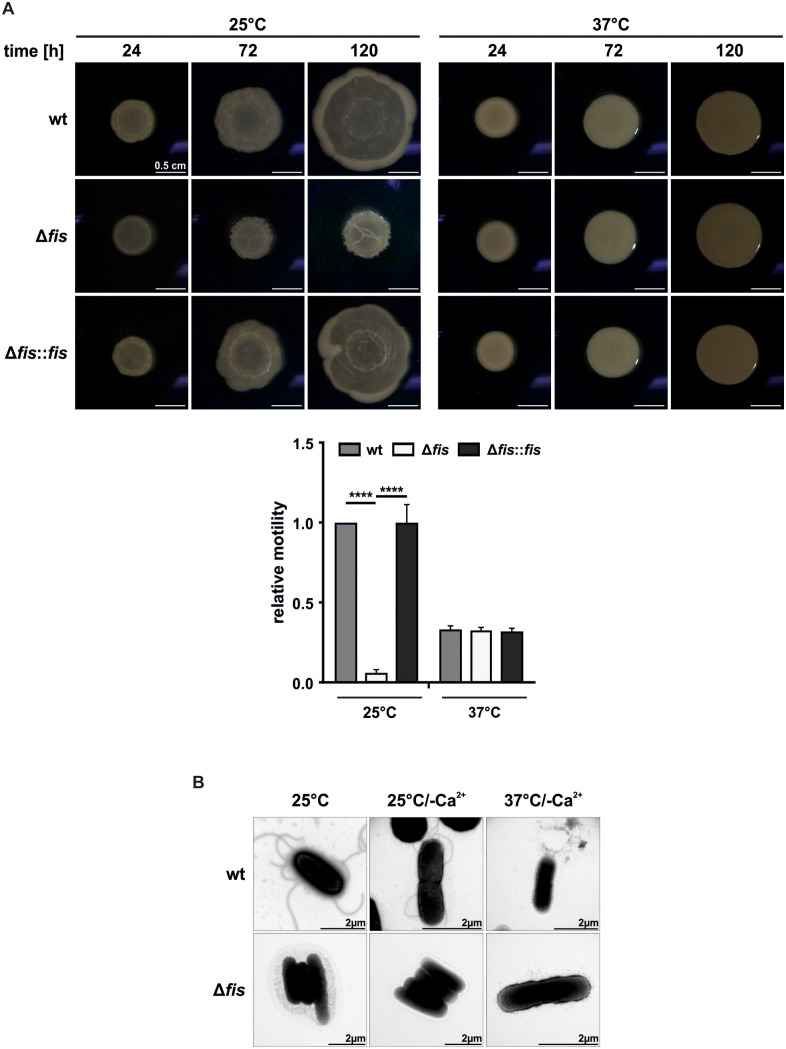
Fis promotes motility by regulating flagellar synthesis. **(A)** Swarming motility assay of *Y. pseudotuberculosis* wt, the *fis* mutant, and the complemented strain (Δ*fis::fis*). Bacterial strains were grown to an OD_600_ of 0.5 at 25°C and a 5 µL droplet was spotted on semi-solid (0.3%) agar plates. Plates were incubated at 25 and 37°C, and colony diameters were measured after 24, 72, and 120 hours. Motility was calculated as the difference in colony diameter between the first and last time points and normalized to the wt incubated at 25°C (lower panel). The data represent the mean and SD of three independent biological replicates. Significance was tested by one-way ANOVA (****p < 0.0001). **(B)** Transmission electron microscopy with negative staining of the wt and *fis* mutant grown at 25 and 37°C in standard or secretion-induced LB medium (–Ca^2+^) to an OD_600_ of 0.5.

In *Yersinia,* motility-associated genes are primarily upregulated at moderate temperatures, facilitating flagellum-mediated motility crucial for survival in free-living environments and for the initial stages of host infection [50,57,58]. Upon host entry, as the temperature rises, this motility program is suppressed [40,59]. However, our transcriptomic analysis revealed that *fis* deletion led to a significant downregulation of nearly all motility-related genes at 25°C (Fig 3A). This correlated with a motility defect, as the *fis* mutant exhibited drastically reduced movement on semi-solid (0.3%) agar plates at 25°C (Fig 4A). This phenotype could be fully restored in the *fis*-complemented strain.

As previously reported, at 37°C, the temperature-dependent motility-associated genes in clusters II and III, including *fliA* encoding the FliA sigma factor, are transcriptionally inactive, resulting in a non-motile phenotype due to the absence of intact flagella [57,60,61]. Consistent with these studies, our RNA-seq data and qRT-PCR analyses showed strong downregulation of class II and III flagellar genes at 37°C (S3 Fig and S3 Table), and all tested strains exhibited a non-motile phenotype at this temperature (Fig 4A).

Electron microscopy further confirmed that the non-motile phenotype of the *fis* mutant at 25°C was due to the absence of flagella under all tested conditions (Fig 4B). In contrast, the wt strain retained flagella at 25°C but lost them at 37°C in a calcium-depleted (–Ca^2+^) medium, a condition that mimics host cell contact under laboratory conditions and induces type III secretion [44,62].

Collectively, these findings demonstrate that Fis serves as a positive regulator of *Yersinia* motility, particularly at ambient temperatures. This aligns with its established role in other Gram-negative bacteria [16,19,47,63,64].

### Growth impairment of the *fis* mutant is a result of permanent secretion

**Fig 5:**
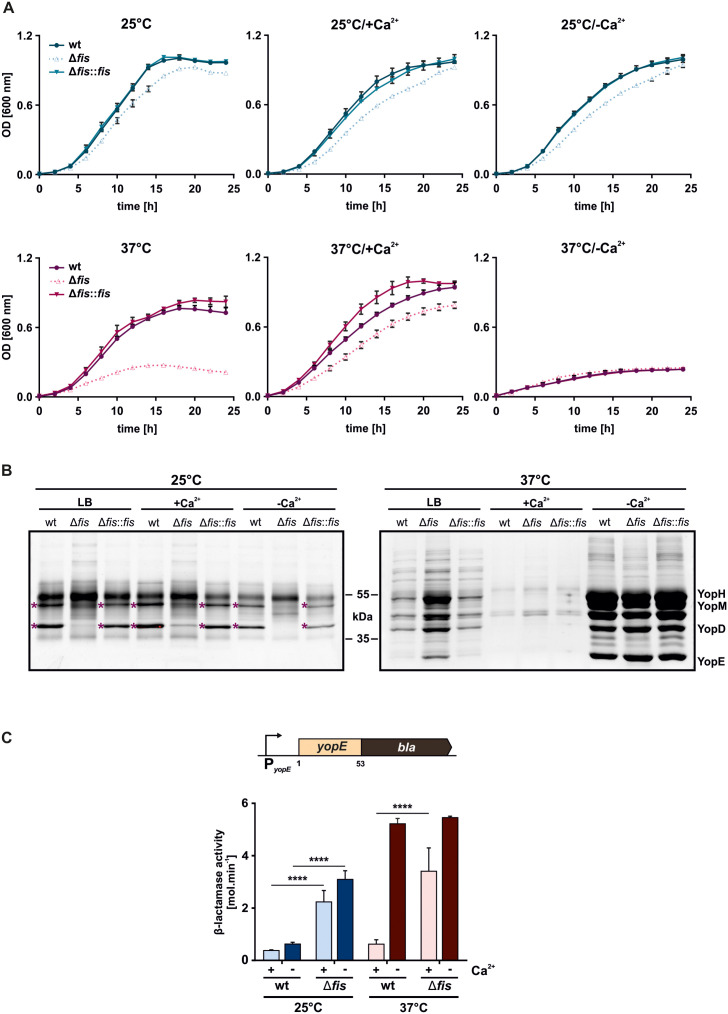
Growth impairment and uncontrolled secretion of the *fis* mutant. **(A)** Growth of *Y. pseudotuberculosis* wt, the *fis* mutant, and the complemented strain (Δ*fis::fis*) in standard LB or T3SS non-secretion (+Ca^2+^) or secretion (–Ca^2+^) medium at 25 and 37°C. Optical density was measured at 600 nm over 24 hours. Error bars indicate SD of technical triplicates of three independent biological replicates. **(B)** Visualization of secreted proteins by SDS-PAGE using SYPRO Ruby staining. Bacterial strains were grown in standard LB or non-secretion (+Ca^2+^) or secretion (–Ca^2+^) medium at 25°C for 1 hour and then for another 3 hours incubated at 25°C or shifted to 37°C. Secreted proteins from culture supernatants were TCA-precipitated and separated by SDS-PAGE. White asterisks indicate protein bands detectable only in the wt and complemented strain. **(C)** Quantification of plasmid-encoded YopE-β-lactamase (schematic illustration) secretion. The fusion construct was expressed in *Y. pseudotuberculosis* wt and *fis* mutant. Strains were grown in a non-secretion (+Ca^2+^) medium at 25°C for 1 hour and then for another 2 hours at 25°C or shifted to 37°C. The cultures grew without induction (+Ca^2+^) or with secretion induction by adding 10 mM EGTA and 20 mM MgCl_2_ (–Ca^2+^) for another 2 hours. Culture supernatants were mixed with 2 mM nitrocefin, and β-lactamase activity was measured as described in Material and Methods. Data represent mean and SD from three independent biological replicates. Significant differences were determined using three-way ANOVA with Šídák’s test (****p < 0.0001).

The reciprocal relationship between reduced motility and induced T3SS/*yop* gene expression is a hallmark of *Yersinia* under virulence-relevant conditions [29,30,50]. Our transcriptomic data revealed that *fis* deletion triggers this expression shift even at 25°C (Fig 3A) when T3SS/*yop* gene expression should be off. To investigate the physiological consequences of this dysregulation, we compared the growth behavior of the *fis* mutant with the wt and the *fis-*complemented strain in LB medium as well as non-secretion (+Ca^2+^) and secretion (–Ca^2+^) media at 25 and 37°C. At 25°C, the *fis* mutant displayed a slight growth delay in all tested media, which was fully rescued in the complemented strain (Fig 5A). Notably, at 37°C, the *fis* mutant exhibited severe growth restriction in LB medium, like the growth inhibition of all strains in secretion (–Ca^2+^) conditions. Growth of the mutant could be partially restored in the presence of calcium, mimicking non-secretion conditions (Fig 5A).

Since growth inhibition of *Yersinia* in a secretion-inducing medium is associated with the continuous secretion of Yop effectors [65–67], we analyzed the amount of proteins secreted into the media under the same conditions as in the growth experiments. SDS-PAGE analysis of the culture supernatants, followed by SYPRO Ruby staining, revealed that at 25°C, the *fis* mutant showed altered secretion patterns compared to the wt and complemented strain (Fig 5B). In addition to these differences, specific protein bands (~40 and ~50 kDa, indicated by asterisks in Fig 5B) were exclusively detected in the wt and complemented strain, but were absent in the *fis* mutant. These bands presumably are extracellular components of the flagellum [43]. At 37°C, all strains exhibited increased secretion levels, particularly in calcium-depleted media (LB and –Ca^2+^). Consistent with the observed growth impairment of the *fis* mutant in these two conditions (Fig 5A), this strain secreted considerably more proteins compared to the wt and complemented strain (Fig 5B), suggesting a link between growth restriction and premature Yop secretion in the absence of Fis.

To demonstrate that plasmid-encoded factors directly contribute to the growth defect of the *fis* mutant, we analyzed the growth rates of strains cured of the virulence plasmid pYV under different conditions. While plasmid curing had only a modest effect at 25°C (S5A Fig), loss of pYV markedly restored the growth rate of the *fis* mutant at 37°C in LB medium, as well as that of all strains under secretion (–Ca^2+^) conditions (S5B Fig).

In addition, we quantified specific Yop secretion by a reporter export assay, which measures the export of β-lactamase fused to the first 53 amino acids of the effector protein YopE (YopE-Bla) (Fig 5C). This construct was expressed under the control of the *yopE* promoter, ensuring T3SS-specific secretion [65,67]. Bacterial cultures were first grown in non-secretion medium (+Ca^2+^) to allow T3SS assembly, followed by calcium chelation to induce secretion at 25 and 37°C (–Ca^2+^). YopE-Bla secretion was then quantified by measuring β-lactamase activity by the nitrocefin assay in the supernatants. Given the reduced calcium sensitivity observed in the *fis* mutant (Fig 5A-B), assays were also performed without induction (Fig 5C). In line with the growth impairment and altered secretion, the *fis* mutant exhibited significantly increased β-lactamase activities, or more precisely, increased Yop secretion, under all tested conditions. As expected, the wt showed β-lactamase activity only when calcium was depleted.

### Fis-dependent *lcrF* repression under environmental conditions

**Fig 6:**
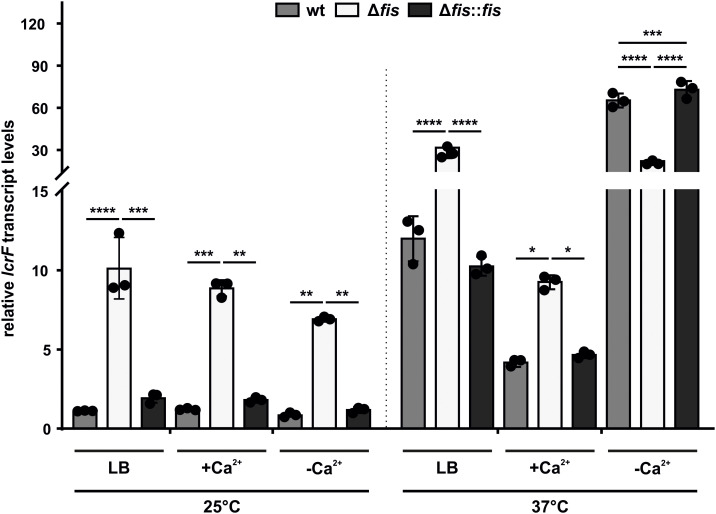
Fis represses *lcrF* expression in response to temperature. Comparison of *lcrF* transcript levels under standard (LB), non-secretion (+Ca^2+^), and secretion (–Ca^2+^) conditions at 25 and 37°C. Samples of *Y. pseudotuberculosis* wt, *fis* mutant, and complemented strain (Δ*fis::fis*) were taken at an OD_600_ of 0.5, followed by RNA isolation and qRT-PCR. Transcript levels were normalized to the amount of *lcrF* in the wt grown at 25°C in standard LB medium and to the reference genes *nuoB* and *hrpA*. Data represent the mean and SD of three biological replicates. Statistical significance was tested using two-way ANOVA followed by Tukey’s test (****p < 0.0001).

Transcription of T3SS and Yop genes is driven by the virulence master regulator LcrF in response to temperature and host cell contact [40]. In our RNA-seq data, *lcrF* was significantly upregulated in the *fis* mutant (Fig 3). To validate these data and assess how *fis* deletion affects *lcrF* expression under different physiological conditions, we performed comparative qRT-PCR analysis using RNA extracted from cultures grown in LB medium (as in the RNA-seq) or under non-secretion (+Ca^2+^) and secretion (–Ca^2+^) conditions at 25 and 37°C. Deletion of *fis* led to consistently elevated *lcrF* transcript levels at 25°C under all conditions (Fig 6). At 37°C, all strains showed the expected induction of *lcrF* under calcium-depleted conditions. In contrast, the *fis* mutant was less responsive to calcium availability and exhibited elevated *lcrF* transcript even in LB (Fig 6). Notably, the *lcrF* transcript levels of the *fis* mutant under non-secretion conditions (+Ca^2+^) were comparably high at both temperatures, indicating that the absence of Fis derepresses baseline *lcrF* expression.

These results clearly demonstrate that Fis acts as a crucial repressor of *lcrF* transcription under environmental conditions in a temperature-dependent manner, which agrees with the secretion phenotypes observed in this study, where *fis* deletion resulted in inappropriate Yop secretion under non-inducing conditions (Fig 5B-C).

### *fis* deletion triggers Yop secretion already at 25°C

**Fig 7:**
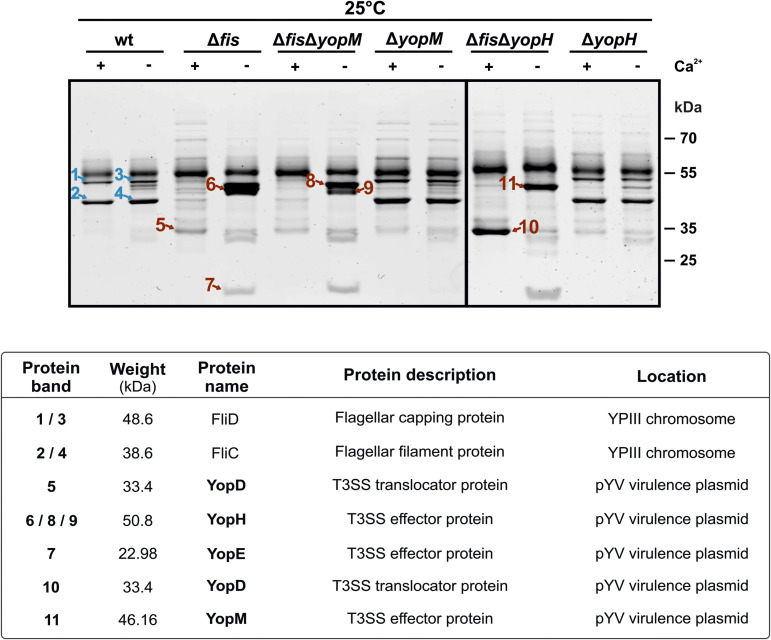
Secretion of *Yersinia* effector proteins at 25°C in the absence of Fis. Identification of the secreted proteins from *Y. pseudotuberculosis* wt and various deletion mutants. Bacterial strains were grown at 25°C in non-secretion (+Ca^2+^) or secretion (–Ca^2+^) medium for 4 hours. Proteins from the culture supernatants were TCA-precipitated and separated by SDS-PAGE and SYPRO Ruby stained. The indicated protein bands were excised from the gels and subjected to trypsin digestion and mass spectrometry. The identity of the proteins is shown in the table.

The experiments described above revealed significant upregulation of virulence plasmid-encoded genes and increased secretion at 25°C in the *fis* mutant. To identify secreted proteins at this temperature, we used high-resolution mass spectrometry. Eleven excised protein bands from SDS-PAGE gels loaded with culture supernatants of the wt and various deletion mutants were subjected to LC-MS analysis (Fig 7). Notably, in *fis* mutants (Δ*fis*, Δ*fis*Δ*yopM,* Δ*fis*Δ*yopH*), all identified proteins corresponded to T3SS effector proteins, demonstrating that loss of Fis leads to inappropriate Yop secretion at low temperature. As expected, YopM and YopH bands were absent in the respective mutants (Δ*fis*Δ*yopM* and Δ*fis*Δ*yopH*). In contrast, the wt did not secrete Yops at 25°C. Instead, the excised bands were identified as flagellin proteins FliC (flagellin) and FliD (filament capping protein), which are continuously secreted during flagellum assembly [68–71]. These findings show that *fis* deletion disrupts the temperature-dependent repression of *yop* gene expression and results in Yop secretion at 25°C.

In line with the proteomic data, native YopE secretion by the *fis* mutant was further confirmed by Western blot analysis, which detected YopE secretion under secretion conditions (–Ca^2+^) already at 25°C. Importantly, YopE secretion was abolished in both the *lcrF* mutant and the *fis*-*lcrF* double mutant, demonstrating LcrF, and hence T3SS dependency (S6 Fig).

**Fig 8:**
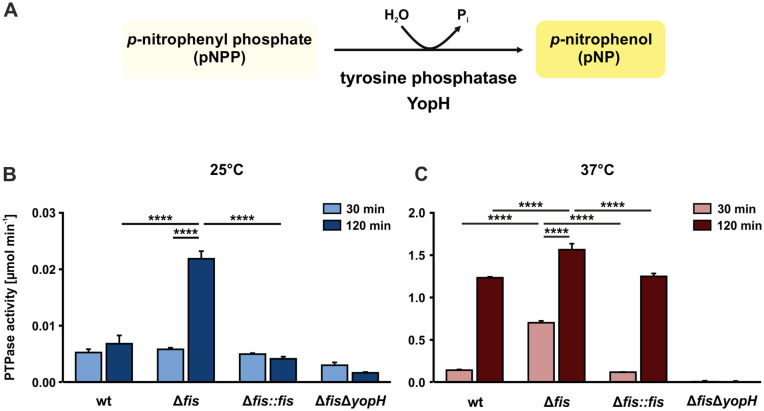
Elevated Yop secretion in the *fis* deletion at 25°C. **(A)** YopH-catalyzed hydrolysis of *p*–nitrophenyl phosphate (pNPP) into the yellow-colored *p*-nitrophenol (pNP). **(B-C)** *Yersinia* secretion assays were conducted at 25 and 37°C with *Y. pseudotuberculosis* wt, *fis* mutant, and complemented strain (Δ*fis::fis*), as well as the *fis*-*yopH* double mutant (Δ*fis*Δ*yopH*) as a negative control. Bacteria were grown at 25°C for 1 hour and incubated for another 2 hours at 25 **(B)** or 37°C **(C)** in the non-secretion medium. Secretion was activated by adding 10 mM EGTA and 20 mM MgCl_2_ and the cultures were incubated for an additional 30 or 120 minutes. Culture supernatants were used for the PTPase assay as described in Material and Methods. Data represent the mean and SD from three independent biological replicates. Significant differences were determined using two-way ANOVA followed by Tukey’s test (****p < 0.0001).

Finally, to assess whether the secreted effector proteins are functionally active, we quantified YopH phosphotyrosine phosphatase (PTPase) activity using a PTPase assay (Fig 8A). YopH, a 51 kDa protein consistently secreted by the *fis* mutant at 25°C (Fig 7), is known for its PTPase activity [72]. After 30 minutes of EGTA/MgCl_2_ addition at 25°C, PTPase activity in the *fis* mutant was comparable to that of the wt and complemented strain (Fig 8B). However, after 2 hours, approximately 3-fold higher activity was measured in the *fis* mutant but not the other strains, indicating continued YopH secretion at 25°C. At 37°C, all strains except for the negative control (Δ*fis*Δ*yopH*) exhibited temperature-dependent induction of YopH secretion (Fig 8C; note the different scale from Fig 8B). Remarkably, the *fis* mutant showed a 4-fold increase in PTPase activity within 30 minutes after EGTA/MgCl₂-mediated induction compared to the wt and complemented strain (Fig 8C). As expected, prolonged incubation at 37°C led to significantly higher YopH secretion and activity in all strains.

Together, our results revealed an uncontrolled Yop secretion in the *fis* mutant under normally non-inducing conditions at 25°C, suggesting a potential influence on virulence, which we tested in the next set of experiments.

### *fis* deletion impairs phagocytosis by macrophages

Physical contact between the T3SS needle and the host cell membrane within the host triggers Yop translocation, which primarily disrupts phagocytosis to evade immune responses [53,54,73]. Given the enhanced T3SS/*yop* gene expression and Yop secretion in the *fis* mutant compared to the wt, we investigated the consequences on bacteria-host interaction and pathogenicity.

**Fig 9:**
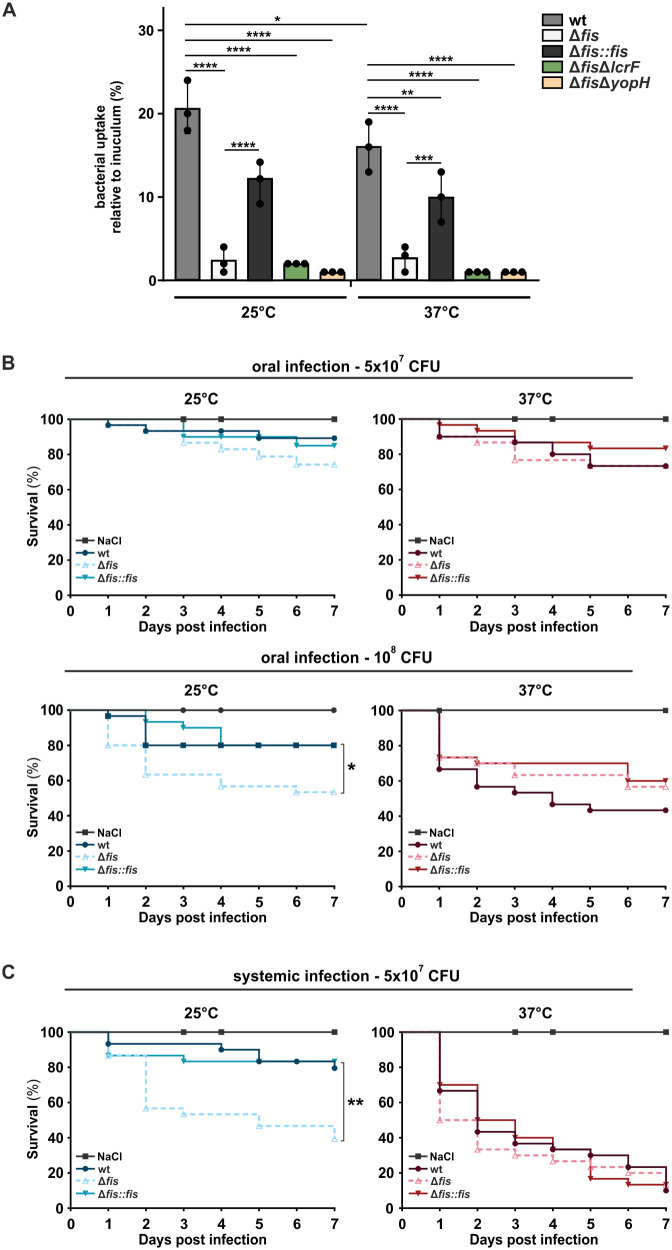
Fis is essential for the inhibition of virulence at low temperatures. **(A)** Macrophage infection assays with *Y. pseudotuberculosis* wt, *fis* mutant, and complemented strain (Δ*fis::fis*) as well as *fis* double mutants (Δ*fis*Δ*lcrF* and Δ*fis*Δ*yopH*) at 25 and 37°C. Bacterial strains were grown in LB medium to an OD_600_ of 0.5 at 25°C and added to human THP-1 macrophages at an MOI of 50. After 1 hour of incubation at 25 or 37°C, extracellular bacteria were removed by washing and treatment with gentamicin. After an additional hour, intracellular bacteria were determined as described in Material and Methods. Data represent the mean and SD from three independent biological replicates relative to the input inoculum. Significant differences were assessed using the two-way ANOVA followed by Tukey’s multiple comparisons test (****p < 0.0001). **(B-C)** Survival of *Galleria mellonella* larvae infected with *Y. pseudotuberculosis* wt, *fis* mutant, and complemented strain (Δ*fis::fis*). Bacterial strains were grown as in (A). Larvae were infected orally (B) with either 5x10^7^ CFU (upper panel) or 10^8^ CFU (lower panel), or systemically injected with 5x10^7^ CFU into the last proleg (C). Control larvae were treated with isotonic saline solution (0.9% NaCl). Following infection, larvae were incubated at 25 or 37°C, and larval survival was monitored daily for 7 days after infection. Data represent three independent biological replicates, with 30 larvae per group in each replicate. Statistical significance of survival differences was determined using the Log-rank (Mantel-Cox) test and Gehan-Breslow-Wilcoxon test (*p < 0.05, **p < 0.01).

To assess whether *fis* deletion impacts bacteria-phagocyte interactions, we performed macrophage infection assays with the *Y. pseudotuberculosis* wt, *fis* mutant, and complemented strain. Bacteria were pre-incubated at 25°C to mimic environmental pre-infection conditions, added to macrophages at an MOI of 50, and incubated at either 25 or 37°C. Intriguingly, macrophages infected with the *fis* mutant exhibited a significantly reduced number of phagocytosed bacteria than those infected with the wt and complemented strain (Fig 9A). This reduced uptake is likely linked to the altered surface properties of the *fis* mutant, including the absence of flagella (Fig 4) and/or the downregulation of *inv* (Fig 3), which encodes the invasin protein required for host-cell attachment and internalization [32,74,75]. To support this hypothesis and to evaluate whether deregulated T3SS activity in the *fis* mutant contributes to this phenotype, we included additional *fis* mutants (Δ*fis*Δ*lcrF* and Δ*fis*Δ*yopH*) in the assay (Fig 9A). All *fis* mutants displayed similarly reduced phagocytosis rates at both temperatures, indicating that neither LcrF-dependent Yop secretion nor YopH-mediated anti-phagocytic activity is responsible for the defect [40,76,77]. Thus, the diminished uptake of the *fis* mutant most likely reflects impaired invasion-mediated entry rather than T3SS-dependent interference with phagocytosis [32,78]. An alternative explanation for the reduced bacterial uptake could be reduced intracellular survival of the *fis* mutant as a result of increased sensitivity to host defense mechanisms, such as oxidative stress responses [23]. However, our RNA-seq data did not reveal any clear evidence of dysregulation in ROS detoxification or other pathways indicative of enhanced intracellular susceptibility.

### *fis* deletion increases pathogenicity in a *Galleria mellonella* virulence model

Finally, we investigated whether the absence of Fis shifts *Yersinia* toward a virulent state at low temperatures. To test this, we used *Galleria mellonella* larvae as a physiologically relevant infection model, which, unlike mammalian models, allows infection studies across a broad temperature range [79,80]. To mimic the natural infection route, larvae were orally infected with two different doses, and survival was monitored for 7 days at 25 or 37°C. At 25°C, larvae infected with the *fis* mutant consistently exhibited a slightly reduced survival rate compared to those infected with the wt or complemented strain, although this effect was not statistically significant at the lower infection dose (5x10^7^ CFU) (Fig 9B). However, when the infection dose was increased (10^8^ CFU), the *fis* mutant displayed significantly enhanced virulence at 25°C, whereas increasing the dose of the wt or complemented strain had no detectable effect on larval survival (Fig 9B).

For systemic infection, we used a dose of 5 × 10^7^ CFU, as this amount resulted in a gradual decline in larval survival over 7 days at 37°C (Fig 9C). Systemic infection exacerbated the inappropriate activation of virulence in the *fis* mutant at 25°C. Larvae infected with the *fis* mutant exhibited a markedly higher mortality rate compared to the oral infection (Fig 9C). In contrast, wt and complemented strains remained attenuated at 25°C regardless of infection route or dosage (Fig 9B-C). At 37°C, *fis* mutant infection accelerated larval mortality; however, overall survival curves did not significantly differ between the tested strains (Fig 9C).

Together, these findings highlight the essential role of Fis in maintaining the free-living state of *Yersinia* at environmental temperatures, particularly by ensuring adhesion/invasion production and preventing premature Yop secretion at low temperatures outside the host.

## Discussion

The interplay between various transcriptional and post-transcriptional mechanisms enables bacteria to precisely control gene expression in response to environmental shifts encountered during host entry and exit [67,81,82]. Integrated into these complex regulatory networks are NAPs that play a pivotal role in coordinating virulence programs and adjusting bacterial physiology and virulence [83,84]. In this study, we investigated the global regulatory function of the NAP Fis in *Y. pseudotuberculosis* at two physiologically relevant temperatures. Our findings reveal that Fis acts as a pleiotropic regulator, balancing motility and pathogenesis in response to environmental cues. Specifically, Fis prevents the premature activation of immune defense genes, ensuring that *Yersinia* remains in an early infection state at moderate temperatures and facilitating the transition to a pathogenic state upon host entry by promoting host cell contact and invasion.

### Signal-dependent switch to a host defense state in *Yersinia*

A temperature upshift to 37°C serves as a reliable cue signaling entry into a warm-blooded host. In *Yersinia*, mammalian body temperature triggers a cascade of regulatory events that induce virulence factors important to counteract the host immune defense, among them the plasmid pYV-encoded T3SS and its effector proteins, the Yops [29]. Yops are translocated via the T3SS upon host cell contact to subvert innate immune responses and facilitate dissemination into deeper tissues [44,45,53,85]. Importantly, premature or inappropriate activation of these virulence factors imposes a considerable metabolic burden, halts bacterial growth, and increases the risk of immune detection [66,86,87]. To avoid this, *Yersinia* employs several thermo-responsive mechanisms that promote initial host-cell contacts immediately after host entry while mediating a rapid switch to counteract host immune defenses at the following stages of infection [29]. A key player in this system is LcrF, the virulence master regulator that drives the transcription of T3SS operons at 37°C [40,88]. Expression of *lcrF* itself is tightly regulated at both transcriptional and post-transcriptional levels in a temperature-dependent manner [39,67,89]. At ambient temperatures, the global histone-like protein YmoA forms a protein complex with H-NS, binding to the *lcrF* promoter and preventing its transcription [36,37,39]. This repression is enhanced by the topological state of DNA at lower temperatures, which increases YmoA-H-NS binding affinity [56,90,91]. Additionally, translation of residual *lcrF* transcripts is blocked by an RNA thermometer (RNAT) that sequesters the ribosome-binding site in a stable hairpin structure [39, 92]. Upon temperature shift to 37°C, YmoA undergoes conformational changes, reducing its ability to form a complex with H-NS, thus triggering its proteolytic degradation [37,56,93]. Simultaneously, the RNAT undergoes thermally induced melting, exposing the RBS and enabling *lcrF* translation. The resulting LcrF protein then activated T3SS operons, ensuring timely virulence induction upon host entry [39].

Beyond temperature regulation, Yop secretion is also controlled by host cell contact, which can be mimicked by calcium depletion under laboratory conditions, a phenomenon known as low calcium response (LCR) [54,94]. This tightly regulated process involves multiple feedback inhibition mechanisms. Key players include YopD, a T3SS translocon protein, and its chaperone LcrH [95,96]. In the absence of host contact, intercellular accumulation of YopD and LcrH promotes *lcrF* repression and controlled degradation of untranslated *yop* mRNA by the degradosome complex, thereby preventing premature Yop synthesis [89,96]. Upon host contact, YopD secretion relieves this repression and enables Yop production and secretion [97]. Another critical regulator of calcium-dependent secretion is YopN, which acts as a gatekeeper of the T3SS secretion channel [62]. Under non-inducing conditions, such as high calcium levels or lack of host contact, YopN remains intracellular, preventing the premature release of Yops [98]. However, upon calcium depletion, YopN is secreted first, allowing the subsequent release of other effectors [86]. Additionally, YopN production is regulated by an RNAT, providing an extra checkpoint to ensure that Yop secretion occurs only under appropriate conditions [87].

### Fis serves as another virulence switch

Despite the numerous regulators described above, yet another global player is involved in *Yersinia* virulence, the NAP Fis. This small protein is well-known for its role in stress responses, motility, secondary metabolism, and virulence in Gram-negative bacteria [22,23,99,100]. In pathogens like *S. enterica, Shigella,* enteroinvasive *E. coli,* and *P. aeruginosa*, Fis has been shown to enhance T3SS-associated virulence [16,20,22,63,101]. In the present study, we uncovered a critical role for Fis in *Y. pseudotuberculosis,* acting as a central modulator in maintaining the temperature-dependent regulatory balance that promotes initial cell contact while inhibiting the activation of late-stage T3SS/*yop* genes, particularly at moderate temperatures.

**Fig 10:**
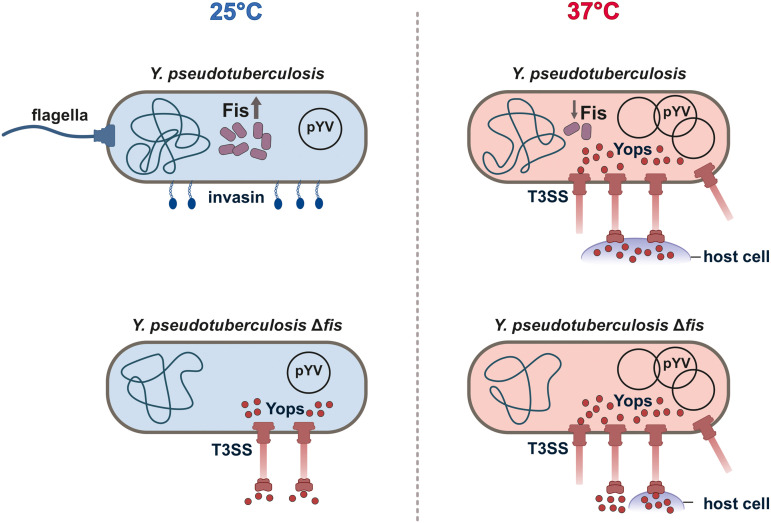
Fis regulates the balance between motility, cell adhesion/invasion, and host immune defense in *Y. pseudotuberculosis* in response to temperature.

Specifically, we found that Fis prevents the transcription of nearly all plasmid-encoded T3SS–associated genes, particularly at 25°C and during the early growth phases at 37°C. In contrast, *fis* deletion resulted in a strikingly inverted expression profile compared to the wt, characterized by strong downregulation of motility-related genes and *inv*, which encodes the invasin protein, whereas T3SS/*yop* genes were significantly upregulated and showed premature secretion under non-secretion conditions. These findings position Fis as a critical repressor that prevents the untimely induction of virulence (Fig 10).

At 25°C, high levels of Fis promote the production of flagella and invasin while suppressing T3SS/*yop* genes, thereby maintaining the bacterium in an environmentally adapted, motile state. When exposed to host conditions at 37°C, reduced Fis levels together with increasing virulence plasmid copy number alleviate this repression, shifting the regulatory balance toward host-defense mechanisms, including repression of motility and induction of the T3SS and Yop secretion upon cell contact.

Despite significant upregulation of T3SS *ysc*/*yop* genes in the *fis* mutant at 25°C, Yop secretion levels did not reach those observed in the wt at 37°C. This is expected, as several additional regulatory layers restrict T3SS activity at lower temperatures. As described above, besides the global repressors YmoA and H-NS at the transcriptional level, several RNATs act as critical post-transcriptional checkpoints to prevent premature T3SS assembly and activity at lower temperatures. In addition to the RNATs upstream of the *lcrF* and *yopN* mRNAs, two RNATs repress translation of *yscT* and *yscJ*, which encode structural components essential for the initial assembly of the injectisome [39,87,102].

Another intriguing finding was the reduced sensitivity of the *fis* mutant to calcium, which resulted in Yop secretion even in the presence of calcium (Fig 5B-C and Fig 7). This suggests that Fis plays a role in regulating the low calcium response and cell-contact-dependent secretion. One potential target of this regulation is YopD, as we observed a negative effect of Fis on *yopD* expression and identified YopD as a protein secreted at 25°C in the *fis* mutant (Fig 7). Given the known regulatory role of YopD in regulating *lcrF* mRNA stability and inhibiting T3SS secretion under non-induced conditions [67,89], we speculate that the YopD secretion at 25°C in the *fis* mutant may stabilize *lcrF* transcripts and thereby induce T3SS expression.

Temperature-dependent increases in virulence plasmid copy number enhance T3SS gene dosage at 37°C [55], but this effect was not observed in the *fis* mutant at 25°C (S4 Fig). Previous studies have shown that YmoA, together with H-NS, as well as the YopD-dependent plasmid replication system (RepA, CopA/B), are key modulators of pYV copy number [56,103]. However, our transcriptomic data showed that expression of *ymoA* and *hns* was unchanged in the *fis* mutant, and despite the strong upregulation and premature secretion of YopD, neither *copA* nor *copB* showed a significant decrease in expression (S2 Dataset). Based on this knowledge, we hypothesize that our results are more consistent with a direct regulatory role of Fis as a NAP that modulates the transcriptional state of the virulence plasmid through its DNA-binding and structural effects, which need to be elucidated in future studies.

### Fis coordinates virulence and motility

The upregulation of virulence genes in the *fis* mutant at 25°C was accompanied by a downregulation of motility and chemotaxis genes (Fig 10), resulting in a non-flagellated, non-motile phenotype. Additionally, the genes *rovA* and *inv*, which encode factors essential for *Yersinia* survival in its free-living state at 25°C and for rapid dissemination in the host following ingestion [50,74,104], were also downregulated. This suggests that Fis plays a critical role in regulating motility and invasion in *Yersinia* prior to the loss of Fis-dependent suppression of antiphagocytic virulence factors after host uptake (Fig 10). Interestingly, while the negative effect of Fis on T3SS/*yop* genes seems to be unique to *Y. pseudotuberculosis*, its positive impact on flagellar assembly and motility is conserved across other species [18,63,64].

Cross-regulation between motility- and T3SS-associated genes is common in bacterial pathogens [57,105,106]. Upon host entry, flagellated enteropathogenic *Yersinia* utilize motility to disseminate within the host until the elevated temperature represses flagellar gene expression and triggers the production of the T3SS, rendering the bacteria non-motile at 37°C (Fig 10) [57,60]. As reported previously and based on our transcriptomic data (S3 Table), temperature-dependent regulation of motility is mainly mediated by class II flagellar genes, including *fliA*, *flhA*, *flhB*, *flgM*, *flgK*, and *flgE*, with FliA, a sigma factor, playing a central role. FliA positively regulates class III genes, such as flagellin operons at 25°C, and is likely a negative regulator of *lcrF* [57,59]. It is plausible that the *fis* mutant interprets the downregulation of flagella and adhesion/invasion genes as a signal resembling the late stages of infection, during which the T3SS is required. This, in turn, leads to the premature induction of the T3SS and enhanced Yop secretion, regardless of environmental signals.

While Fis reciprocally regulates T3SS- and motility-related gene expression in *Yersinia*, the situation is markedly different in *S. enterica* serovar Typhi. Here, mutants in genes encoding flagellar regulatory proteins significantly reduce the expression of the T3SS regulon and diminish effector secretion, suggesting a positive cross-talk between these motility and T3SS pathways [50,107]. This raises the question of why Fis has a negative impact on virulence in *Yersinia*. Previous studies in *Salmonella* demonstrated that Fis directly binds to the *flhD* promoter, activating the master regulator of the flagellar hierarchy [63]. Because the flagellar system and *Salmonella* pathogenicity island (SPI)-1 are co-expressed and co-regulated, Fis thereby promotes both motility and T3SS expression during the invasion stage of *Salmonella* infection. These regulatory differences reflect the distinct infection strategies of the two pathogens. In *Salmonella*, effectors translocated by SPI-1-encoded T3SS together with flagellar are required for host-cell invasion and for survival within macrophages rather than extracellular anti-phagocytic functions, as in *Yersinia* [15,48,50,108]*.* Therefore, the regulatory hierarchy upstream of T3SS genes is tuned to invasion-linked cues, whereas *Yersinia* must avoid premature activation of its T3SS outside the host [50,109]. Despite differences in the biological outcomes, the overall regulatory overlap of Fis in enteric bacteria supports our conclusion that Fis functions primarily during the initial stages of infection. This is further corroborated by our phagocytosis assays, in which all *fis* deletion strains exhibited comparable deficiencies in uptake by macrophages (Fig 9A).

An additional intriguing question is how Fis remodels gene expression in response to temperature. Both at RNA and protein levels, Fis concentrations are highest at low temperature and low optical density, though the mechanisms regulating these fluctuations are not well understood. As a NAP, Fis’ regulatory function is likely closely linked to DNA structure and topology [110]. Fis is known to function as a topological buffer, contributing to the maintenance of DNA supercoiling in close cooperation with DNA topoisomerase and gyrase [111,112]. In this context, its ability to organize and fold both chromosomes and plasmids may further extend its regulatory role by modulating promoter accessibility across large genomic domains for global chromosome architecture (Fig 10) [113].

Environmental changes, especially temperature fluctuations, can alter DNA structure and topology, thereby affecting the interaction of DNA-binding and bending proteins like Fis [114]. In *Yersinia*, elevated temperatures melt intrinsic DNA bends, particularly on the pYV virulence plasmid, which correlates with the activation of virulence [91].

Given that Fis acts as a topological homeostat, counteracting the negative effects of excessive DNA relaxation or negative supercoiling, we propose a model in which *Yersinia* uses Fis as a central sensor to maintain the DNA topological state for optimal gene expression in response to environmental changes (Fig 10). This is analogous to that of Fis in *S. enterica* serovar Typhimurium and *E. coli* [21,115,116]. Whether Fis binds to different DNA regions at different temperatures or whether it functions as a stress sensor undergoing temperature-induced conformational changes, like YmoA [56], remains an intriguing area for future research, employing genome-wide DNA-binding techniques such as chromatin-affinity purification and sequencing (ChAP-seq; [117]), as well as biophysical structural biology approaches.

## Materials and methods

### Bacterial strains, plasmids, and growth conditions

The bacterial strains, plasmids, and oligonucleotides used in this study are listed in S1 and S2 Tables. *E. coli* strains were cultured at 37°C, while *Y. pseudotuberculosis* strains were grown in LB (Lysogeny broth) medium at 25°C unless otherwise specified. For plasmid-carrying bacteria, antibiotics were added at the following concentrations: ampicillin 100 μg/mL, chloramphenicol 20 µg/mL, and kanamycin 50 µg/mL.

### Construction of *Yersinia* mutants and complementation strain

The *Y. pseudotuberculosis* strains used in this study are derived from wild type strain YPlll (NZ_CP009792.1). All deletion strains were generated by homologous recombination using pDM4 plasmid as previously described [118]. Briefly, the *Yersinia* genomic DNA served as a template to amplify desired PCR fragments, containing a 5´- and a 3´-flank of each target gene, using primers listed in S2 Table. Amplified PCR fragments were recombined using splicing by overlap extension PCR (SOE-PCR) [119] and cloned into the SacI-digested pDM4 suicide plasmid. Next, recombinant plasmids were transferred into *E. coli* S17-1 λ-pir competent cells and later introduced into *Y. pseudotuberculosis* YPlll by conjugation. Putative deletion mutants were tested by PCR with internal and external primer combinations and confirmed by DNA sequencing.

For complementation of the *fis* deletion mutant*, fis* was tagged with a C-terminal polyhistidine (His)-tag at its native chromosomal locus using SOE-PCR. The *fis* gene was amplified with the primer pair 5´_fw/5´_rv, generating a ~ 600 bp fragment that included the upstream region of *fis* plus the *fis* coding region, the His-tag, and the stop codon. The ~ 600 bp downstream region of *fis* was amplified with primer pairs 3´_fw/3´_rv of which the forward primer contained the overlap sequence of the upstream fragment. The amplified PCR fragments were cloned into the SacI-digested pDM4 suicide plasmid, followed by transformation in *E. coli* S17-1 λ-pir and subsequent conjugation into *Y. pseudotuberculosis* YPlll *fis* mutant. Correct integration was confirmed by PCR and DNA sequencing.

### Curing of the *Yersinia* virulence plasmid

Cultures were inoculated from overnight pre-cultures grown at 25°C to an OD_600_ of 0.01, and incubated for 24 h at 37°C. Ten-fold serial dilutions (1^00^-10^-4^) were plated onto Congo red-magnesium oxalate (CRMOX) plates and incubated overnight at 37°C [120]. White colonies were selected, re-streaked on LB agar, and the absence of the pYV plasmid was verified by colony PCR.

### RNA isolation and Northern blot analysis

Bacterial cultures were grown under the desired conditions and the RNA isolation of cell pellets was followed by using SDS/hot phenol as described previously [87]. Briefly, a total of 4 mL culture was harvested by centrifugation (5 min, 13000 rpm, 4°C). The supernatant was removed, and pellets were resuspended in 250 µL TE buffer (1 mM EDTA, 10 mM Tris, pH 7.5) and 12.5 µL SDS (10% w/v). Then, 450 µL of pre-heated (60°C) saturated aqua-phenol was added, mixed with the lysate, and incubated for 10 min at 60°C. After incubation, the samples were chilled on ice for 1 hour and centrifuged (1 h, 13000 rpm, 4°C). The aqueous layer was transferred to a Phase Lock Gel (PLG, Heavy) tube (Eppendorf, Hamburg, Germany) with an equal volume of chloroform and centrifuged (5 min, 13000 rpm, 4°C). Finally, RNA was precipitated with ethanol at -20°C. The RNA was recovered by centrifugation (1 h, 13000 rpm, 4°C), the RNA pellets were washed at 4°C with 70% (w/v) ice-cold ethanol two times and then air-dried for 20 min and resuspended in 40 µL nuclease-free water (Carl Roth GmbH, Karlsruhe, Germany).

For Northern blot analyses, total isolated RNA (10 µg) was separated on MOPS agarose gels (1.2%) and transferred onto a nylon membrane by capillary transfer. RNA was hybridized with RNA probes derived from *in vitro* transcription with DIG-labeled nucleotides (Roche, Mannheim, Germany). Luminescence was detected using the substrate CDP star (Roche, Mannheim, Germany) and the ChemiDoc Image System (Bio-Rad).

### Quantitative real-time PCR (qRT-PCR)

qRT-PCR was performed in technical triplicate with RNA isolated from biological triplicates and was carried out with the CFX Connect Real-Time system (Bio-Rad Inc.). Strains were grown under desired conditions to an optical density (OD_600_) of 0.5. Total RNA was then extracted from each sample as described above. Chromosomal DNA residuals were removed using the TURBO DNA-free Kit (Thermo Fisher Scientific) following the manufacturer’s protocol. cDNA synthesis was performed using the iScript cDNA synthesis Kit (Bio-Rad) according to the manufacturer’s protocol with 1 μg RNA per reaction. 2 µL of 1:10 diluted cDNA was mixed with 250 nM of each primer, 5 µL of 2x iTaq Universal SYBR Green Supermix, and 2.5 µL sterile water (Carl Roth). To calculate primer efficiency and determine the linear range of amplification, standard curves were employed. For the calculation of relative *fis* transcript levels, the primer efficiency corrected method was used [121]. The reference genes *nuoB* and *hrpA* served for normalization [29]. Gene-specific primers used for qRT–PCR amplification are listed in S2 Table.

### RNA-sequencing

*Y. pseudotuberculosis* wild type and the *fis* mutant were used for RNA-seq transcriptomics analysis. Both strains were grown in LB medium at 25 and 37°C to the early (OD_600_ 0.5) and late exponential growth phase (OD_600_ 1.5). Total RNA was then extracted from each sample as described above and treated with the TURBO DNA-free Kit (Thermo Scientific, Waltham, USA) according to the manufacturer’s information to obtain pure RNA. Library preparation and sequencing were conducted by Novogene Co., LTD using the Illumina NovaSeq 6000 platform. The sequencing results were aligned with the reference genome of the *Y. pseudotuberculosis* YPlll database (reference genome: NZ_CP009792.1, plasmid pIB1: NZ_CP032567.1). Statistical evaluation was performed as previously reported [122]. Genes with adjusted p–values of 0.001 and a log_2_ fold change of ≤-1.5 or ≥1.5 relative to the wild type were considered as differentially expressed.

### Data access

The high-throughput read data is deposited at the Gene Expression Omnibus (GEO) database under the accession number GSE295938. The comparative transcriptome analyses are given in S1 and S2 Datasets. The comparison analyses of the wt are available with accession no.: GSE270081.

### Quantitative PCR (qPCR) analysis of virulence plasmid copy number

*Y. pseudotuberculosis* strains were cultured overnight in LB medium at 25°C, sub-cultured to an OD_600_ of 0.05, and grown to an OD_600_ of 0.5 either at 25 or 37°C. After incubation, total DNA was extracted using GeneJET Genomic DNA purification Kit (ThermoScientific) following the manufacturer’s protocol. The DNA concentration was measured, and 1 ng/μl of total DNA was used for qPCR, as described in [55]. Briefly, the total DNA was amplified in SensiFAST SYBR No-ROX (Bioline) using three primer pairs for chromosomal genes (*glnA*, *rpoB*, *YPK*_*3178*) and two primer pairs for the plasmid genes (*yscM*, *repA*), listed in S2 Table. qPCR was performed in technical duplicates with DNA isolated from biological triplicates in the Rotor-Gene Q real-time PCR cycler (Qiagen). Two reactions with nuclease-free water without DNA were used as controls. A melting curve was employed to ensure specificity. The plasmid copy number of the strains was calculated as described by [55,67].

### Swarming motility assay

Bacterial strains were grown to an OD_600_ of 0.5 at 25 or 37°C. Subsequently, 5 µL droplets of the bacterial cultures were spotted on the semi-solid agar (0.3%) plates and incubated for five days at respective temperatures. The diameter of the bacterial colony was measured and documented as an indicator of swarming ability.

### Transmission electron microscopy

Bacterial strains were grown in LB or calcium-depleted medium at 25 or 37°C to OD_600_ of 0.5. Immediately after reaching the desired OD, cultures were mixed 1:1 with fixation solution containing 4% formaldehyde and 0.4% glutaraldehyde in D-BPS (pH 7.4). A drop of fixed bacterial culture was applied onto a formvar-coated, carbon-sputtered 200-mesh copper grid. After negative staining with 1% phosphotungstic acid, samples were analyzed at 80 kV using an FEI Tecnai 12 electron microscope (FEI, Eindhoven, Netherlands). Images were acquired with a Veleta 4k CCD camera (Emsis, Münster, Germany).

### Growth experiments

*Y. pseudotuberculosis* strains were cultured overnight in LB medium at 25°C and sub-cultured to an OD_600_ of 0.01 in a final volume of 200 µL in either standard LB medium (1% tryptone, 1% NaCl, 0.05% yeast extract), or in T3SS non-secretion medium (2.5 mM CaCl_2_), or secretion-induced medium (20 mM MgCl_2_, 20 mM sodium oxalate as a calcium chelator) using 96-well plates. The microplates were then incubated at either 25 or 37°C with shaking in a temperate plate reader (TECAN Infinite 200 PRO), followed by OD measurements for about 24 hours.

### *Yersinia* secretion assay

The Yop secretion assay was performed as previously described [102]. For standard *Y. pseudotuberculosis* T3SS induction, bacteria were grown overnight in LB medium and cultured in fresh secretion-inducing medium to an OD_600_ of 0.1. After 1 hour of growth at 25°C, cultures were either shifted to 37°C for at least 4 hours or maintained at 25°C for 4 hours to visualize secretion at ambient temperature. Cultures were then normalized to OD_600_ 0.8 and centrifuged (10 min, 4000 rpm, RT). Proteins in the sterile-filtered supernatant were precipitated overnight at 4°C by adding trichloroacetic acid (TCA) to a final concentration of 10% and harvested by centrifugation (20 min, 4000 rpm, 4°C). The resulting pellets were first resuspended in 0.5 mL of 2% SDS solution, then mixed with 1.5 mL of ice-cold 100% acetone and incubated for 30 min at -20°C. After washing twice with 0.5 mL acetone, pellets were dried for 15 min at 30°C and subsequently resuspended in 2x SDS sample buffer (4% (w/v) SDS, 25 mM EDTA, 2% (v/v) β-mercaptoethanol, 20% (v/v) glycerol, 0.04% (w/v) bromophenol blue, 100 mM Tris, pH 6.8). Samples were boiled for 5 min before running on a 12.5% SDS polyacrylamide gel to separate *Yersinia* effector proteins (Yops).

### Western blot analysis

For the detection of proteins of interest, cell pellets of *Y. pseudotuberculosis* strains were resuspended in 1x SDS sample buffer (2% (w/v) SDS, 12.5 mM EDTA, 1% (v/v) β–mercaptoethanol, 10% (v/v) glycerol, 0.02% (w/v) bromophenol blue, 50 mM Tris, pH 6.8). Resuspended protein samples were heated at 95°C for 5 min, centrifuged (5 min, 13000 rpm), and loaded onto a 12.5% SDS polyacrylamide gel. The gels were either stained with SYPRO Ruby or blotted onto a nitrocellulose membrane using the Bio-Rad Trans-Blot Turbo. Total proteins were visualized by Ponceau S staining (0.01% (w/v) Ponceau S, 1% (w/v) acetic acid). For detection of His-tagged Fis protein, a penta-His HRP conjugate was used (1:4000, QIAGEN, Hilden, Germany). Secreted YopE was detected using a rabbit anti-YopE antibody (1:3,000; Dersch lab), followed by a goat anti-rabbit HRP-conjugated secondary antibody (1:5,000; Bio-Rad, Munich, Germany). Protein signals on the membranes were detected with Immobilon Forte Western HRP substrate (Merck, Darmstadt, Germany) with the ChemiDoc Imaging System (Bio-Rad) and further analyzed with the Image Lab software provided by Bio-Rad.

### β-lactamase assay

*Y. pseudotuberculosis* strains harboring plasmid pMK-*bla* encoding a *yopE*-*bla* fusion were grown overnight in LB medium with kanamycin at 25°C and then cultured in fresh secretion-noninduced medium containing 2.5 mM CaCl_2_ and kanamycin at an OD_600_ of 0.1. Cultures were grown at 25°C for 1 hour, followed by incubation either at 25°C or transferred to 37°C for another 2 hours. When performing induction of Yop secretion, 10 mM EGTA and 20 mM MgCl_2_ were added. After 2 hours, the OD_600_ of each culture was measured and strains were diluted to achieve equivalent optical densities. For each sample, 95 µL supernatant was added to a Sarstedt TC 96-well plate in triplicates. Then, 5 µL per well of β-lactamase substrate solution (2 mM Nitrocefin (Merck) in phosphate-buffered saline) were added. After 1 hour incubation at room temperature, β-lactamase activity was quantified by measuring the increase in absorbance at 486 nm, caused by β-lactamase-catalyzed hydrolysis of Nitrocefin, using a Tecan Infinite 200 Pro photometer. The average β-lactamase activity from three independent experiments was calculated as previously described [67].

### YopH phosphatase (PTPase) activity assay

The phosphatase activity of secreted YopH was measured as previously described [77,123]. *Y. pseudotuberculosis* strains were grown overnight in LB medium at 25°C and then cultured in fresh non-secretion medium containing 2.5 mM CaCl_2_ at an OD_600_ of 0.1. Cultures were grown at 25°C for 1 hour, followed by incubation either at 25°C or transferred to 37°C for an additional 2 hours. Yop secretion was induced by adding 10 mM EGTA and 20 mM MgCl_2_. After 30 and 120 minutes, the supernatants were collected for PTPase activity measurement.

The PTPase assay was conducted at 37°C in a 200 µL reaction volume containing 25 mM *p*–nitrophenyl phosphate (*p-*NPP, Sigma) as the substrate, 40 mM MES (2-(*N*-morpholino) ethanesulfonic acid) pH 5.0 buffer, and 1.6 mM DTT as a reducing agent. The reaction was stopped after 10 min by adding 0.2 M NaOH. The amount of released *p*-nitrophenol was quantified by measuring the absorbance at 405 nm. The PTPase activity was calculated according to the following formula: PTPase activity (µmol/min) = [(A_405nm_ x V_total_)/(ε x 1 cm x t_min_ x V_sample_)]/OD_600nm_, where ε represents the molar extinction coefficient for pNPP, which is 1.8 × 10⁴ M ⁻ ¹ cm ⁻ ¹ [123].

### Protein digestion and mass spectrometry analysis

Protein bands were excised from the gels and destained twice with 400 µL of washing solution (20 mM ammonium bicarbonate in 30% acetonitrile (ACN)) at 37°C and 900 rpm. Cysteines were reduced with 50 µL of 10 mM dithiothreitol in washing solution at 60°C for 60 min and alkylated with 50 mM iodoacetamide in washing solution at 25°C in the dark for 30 min. Gel bands were dried and soaked with 12.5 ng/µL sequencing grade trypsin, and excessive supernatant was discarded after 30 min at 37°C. Following overnight digestion at 37°C, peptides were recovered in an ultrasonic bath for 15 minutes with 40 µL of 0.1% trifluoroacetic acid (TFA) in MS grade water.

For mass spectrometry, 5 µL of peptides were injected into an ACQUITY UPLC I-Class System (Waters) equipped with an ACQUITY UPLC C18 column (Waters, 1.7 μm, 2.1 x 50 mm). Peptides were eluted using a gradient of 0.1% formic acid in MS grade water (solvent A) or acetonitrile (solvent B) with a flow rate of 0.4 mL/min: 0-0.5 min, 1% B; 4.75 min, 40% B; 5 min, 85% B; 5.5 min, 85% B; 5.6 min, 1% B; 7 min, 1% B. The column temperature was set to 40°C. Peptides were eluted online to a Vion IMS-QToF-MS (Waters) mass spectrometer equipped with an ESI source. MSE spectra were recorded in positive sensitivity mode with a scan time of 0.2 s. Nitrogen was used as collision gas with a collision ramp of 28–60 V. The following parameters were used: capillary voltage, 0.8 kV; cone voltage, 40 V; source offset, 80 V; cone gas flow, 50 L/h; desolvation gas flow, 1,000 L/h; source temperature, 150°C; desolvation temperature, 550°C. Leucine enkephalin was recorded as the lock mass.

Protein identification was performed using ProteinLynx Global Server (Waters, Version 3.0.3) with a *Y. pseudotuberculosis* YPIII database (reference genome: NZ_CP009792.1, plasmid pIB1: NZ_CP032567.1). Parameters were set as follows: chromatographic peak width, automatic; MS TOF resolution, automatic; lock mass for charge 1, 556.2771 Da/e; lock the mass window, 0.25 Da; low energy threshold, 50.0 counts; elevated energy threshold, 10.0 counts; peptide and fragment tolerance, automatic; minimum fragment ion matches per peptide, 2; minimum fragment matches per protein, 5; minimum peptide matches per protein, 1; maximum protein mass, 250 kDa; trypsin as the primary digest reagent; no secondary digest reagent; missed cleavages, 1; fixed modifications, carbamidomethyl C; variable modifications, oxidation M; false discovery rate, 4%.

### Analysis of the *Yersinia* phagocytosis by human macrophages

The phagocytosis assay was performed with THP-1 cells according to the method described by [67]. THP-1 cells were seeded at a density of 2x10^5^ cells/well in a 24-well plate and differentiated into adherent macrophages using 50 ng/mL PMA. The medium was replaced with fresh medium 24 hours before infection. Bacteria were cultured in LB medium to an OD_600_ of 0.5 at 25°C and added to macrophages at a multiplicity of infection (MOI) of 50. Infections were carried out at either 25 or 37°C for 1 hour. Afterwards, macrophages were washed with PBS, and fresh medium containing 50 μg/mL gentamicin was added to eliminate the extracellular bacteria. After an additional incubation of 1 hour at the respective temperatures, macrophages were lysed with 0.1% Triton X-100 to release intracellular bacteria. Lysates were plated on LB agar, and bacterial colonies were counted. Bacterial uptake was calculated relative to the initial bacterial inoculum.

### *Galleria mellonella* infection assay

The *Galleria mellonella* infection assay was performed using final instar larvae, each weighing approximately 250–350 mg, obtained from Fauna Topics (Marbach, Germany) and maintained at 25°C in the laboratory. To avoid sampling biases, larvae with any signs of melanization or deformity were excluded. Before all injections, larvae were surface-sterilized with 70% ethanol.

Bacterial cultures were grown overnight in LB medium at 25°C, followed by growth in fresh medium to an OD_600_ of 0.5. Cells were harvested by low-speed centrifugation (2 min, 1000 rpm, 25°C), and resuspended in physiological saline (0.9% NaCl) to the desired concentration.

Infections were administered using a manual microsyringe pump (World Precision Instruments Germany GmbH) to ensure a consistent injection volume and site. For oral infection (force–feeding), 10 µL of the bacterial solution was slowly applied using a fine cannula (33G needle), inserted between the labrum and labium, until the larva completely absorbed the fluid. For systemic infection, 10 µL of the bacterial suspension was injected into the last left proleg of each larva. During infection, larvae were not fed to maintain consistent experimental conditions. Control groups were injected with 10 µL of sterile saline using the same method.

Post-infection, larvae were incubated at 25 or 37°C and monitored daily for survival over 7 days. Mortality was recorded, and Kaplan-Meier survival curves were generated for data analysis. Each experiment was performed in triplicate with a minimum of thirty larvae per group to ensure statistical validity.

### Statistical analysis

Statistical analyses were performed using GraphPad Prism 10.4. Depending on the experimental setup, comparisons between two groups were made using unpaired two-tailed t-tests, while comparisons between multiple groups were performed using one-way, two-way, or three-way ANOVA followed by appropriate post hoc tests.

## Supporting information

S1 Table Bacterial strains and plasmids used in this study. (DOCX)

S2 Table Oligonucleotides used in this study. (DOCX)

S3 Table Temperature-dependent expression of motility-associated genes. (DOCX)

S1 Dataset Comparative RNA-seq analysis wild type (YPIII) versus *fis* mutant. (XLSX)

S2 Dataset Differentially expressed genes of *fis* mutant compared to the wild type identified by RNA-seq analysis. (XLSX)

S1 Fig Transcript abundance of *fis* at 25 and 37°C. (TIF)

S2 Fig Temperature- and growth phase-dependent differentially expressed genes in the *fis* mutant compared to *Y. pseudotuberculosis* wild type. (TIF)

S3 Fig Transcript levels of flagellum-related genes at 25 and 37°C. (TIF)

S4 Fig Determination of virulence plasmid copy number by quantitative PCR (qPCR). (TIF)

S5 Fig Influence of *Yersinia* virulence plasmid (pYV) on growth. (TIF)

S6 Fig Western blot analysis of YopE secretion. (TIF)
