# Supplementary material for: Fis suppresses late-stage virulence gene expression in Yersinia pseudotuberculosis at environmental temperatures
Source: PLoS Pathog. 2026 Mar 25;22(3):e1014105. doi: 10.1371/journal.ppat.1014105 (PMC13046262; doi:10.1371/journal.ppat.1014105)
Supplement: S1 Table — (DOCX) [file ppat.1014105.s001.docx]

**S1 Table.** Bacterial strains and plasmids used in this study.

| **Strain or plasmid** | **Relevant genotype** | **Reference** |
| --- | --- | --- |
| ***Escherichia coli*** |  |  |
| S17-1 *λpir* | RP4-2 Tc::Mu-Km::Tn7 (*λpir*) | [1] |
| ***Yersinia pseudotuberculosis*** |  |  |
| YPIII | pIB1, wild type | [2] |
| YPIII Δ*fis* | pIB1, Δ*fis* | This study |
| YPIII Δ*fis::fis_his_* | *pIB1, Δfis::fis_his_* strain with chromosomal C-terminal his-tagged *fis* | This study |
| YPIII Δ*lcrF* | pIB1, Δ*lcrF* | This study |
| YPIII Δ*fis*Δ*lcrF* | pIB1, Δ*fis*Δ*lcrF* | This study |
| YPIII Δ*yopM* | pIB1, Δ*yopM* | Dersch lab, Münster, Germany |
| YPIII Δ*fis*Δ*yopM* | pIB1, Δ*fis*Δ*yopM* | This study |
| YPIII Δ*yopH* | pIB1, Δ*yopH* | This study |
| YPIII Δ*fis*Δ*yopH* | pIB1, Δ*fis*Δ*yopH* | This study |
| **Plasmids** |  |  |
| pDM4 | *sacBR, oriT*, *oriR6K*, Cm^r^ | [3] |
| pMK-*bla* | HindIII *sycE*-*yopE*_53_-BamHI-NLS-Cre-SalI fragment of pMK4 was replaced by a HindIII *sycE*-*yopE*_53_-BamHI-bla-SalI fragment of pBM53-Bla. Kan^r^ | [4] |
| pTS02 | pGP20, ori pSC101, *lacZ*, AmpR | [5] |
| pBO7411 | pDM4, *fis* (YPK_0452) deletion fragment for generation of *fis* mutants by bacterial conjugation | This study |
| pBO7817 | pDM4, conjugating vector for generation of a chromosomal *fis* complementation | This study |
| pBO6897 | pDM4, *yopH* (pYV00094) deletion fragment for generation of *yopH* mutant by bacterial conjugation | This study |
| pBO6893 | pTS02, ori pSC101*, yscW* promoter(-355 to +12)-‘*lacZ*, Amp^R^ | This study |
