# Supplementary material for: Fis suppresses late-stage virulence gene expression in Yersinia pseudotuberculosis at environmental temperatures
Source: PLoS Pathog. 2026 Mar 25;22(3):e1014105. doi: 10.1371/journal.ppat.1014105 (PMC13046262; doi:10.1371/journal.ppat.1014105)
Supplement: S2 Table — (DOCX) [file ppat.1014105.s002.docx]

**S2 Table.** Oligonucleotides used in this study.

| **Primer** | **Sequence 5’ - 3’** | **Purpose** | **Plasmid** |
| --- | --- | --- | --- |
| *fis*_5’_fw | TTTGAGCTCTGACGTTGAAGATCCGGAC | forward primer to amplify the 5’-flank of *fis* (YPK_0452) for deletion plasmid | pBO7411 |
| *fis*_5’_rv | AGTTCTGTCAGCTCTTTT | reverse primer to amplify the 5’-flank *fis* (YPK_0452) for deletion plasmid | pBO7411 |
| *fis*_3’_fw | AAAAGAGCTGACAGAACTTACTAGTCAGTTAACTTGTTG | forward primer to amplify the 3’-flank of *fis* (YPK_0452) for deletion plasmid | pBO7411 |
| *fis*_3’_rv | TTTGAGCTCTATGAACTTGACGATAAGC | reverse primer to amplify the 3’-flank of *fis* (YPK_0452) for deletion plasmid | pBO7411 |
| *fis*_5’_comp_fw | TTTGAGCTCTAAGTCGCGTTTACGCATGG | forward primer to amplify the 5’-flank and *fis* (YPK_0452) coding region for his-tagged *fis* complementation plasmid | pBO7817 |
| *fis*_5’_comp_rv | TCAGTGATGGTGATGGTGATGGTTCATGCCGTATTTTT | reverse primer to amplify the 5’-flank and *fis* (YPK_0452) coding region for his-tagged *fis* complementation plasmid | pBO7817 |
| *fis*_3’_comp_fw | CATCACCATCACCATCACTGATACTAGTCAGTTAACTTG | forward primer to amplify the 3’-flank and *fis* (YPK_0452) coding region for his-tagged *fis* complementation plasmid | pBO7817 |
| *fis*_3’_comp_rv | TTTGAGCTCAGAGCGTGACCAACGTATC | reverse primer to amplify the 3’-flank and *fis* (YPK_0452) coding region for his-tagged *fis* complementation plasmid | pBO7817 |
| *yopH*_5’_fw | TTTGAGCTCAGCGCCAGACATTCACGA | forward primer to amplify the 5’-flank of *yopH* (pYV0094) for deletion plasmid | pBO6897 |
| *yopH*_5’_rv | GCTTCCCTCCTTAATTAA | reverse primer to amplify the 5’-flank of *yopH* (pYV0094) for deletion plasmid | pBO6897 |
| *yopH*_3’_fw | TTAATTAAGGAGGGAAGCAGAGATATACACCACTTTGCC | forward primer to amplify the 3’-flank of *yopH* (pYV0094) for deletion plasmid | pBO6897 |
| *yopH*_3’_rv | TTTGAGCTCAAGGCCATCTACACCACGA | reverse primer to amplify the 3’-flank of *yopH* (pYV0094) for deletion plasmid | pBO6897 |
| *nuoB*_qRTPCR_fw | GATCCTCTCGAGCAACATG | forward primer to amplify the *nuoB*  transcript for qRT-PCR analysis | - |
| *nuoB*_qRTPCR_rv | TAAAGCAGGTTCCGGCCA | reverse primer to amplify the *nuoB* transcript for qRT-PCR analysis | - |
| *hrpA*_qRTPCR_fw | CTGCTGGCTGAAATTCAG | forward primer to amplify the *hrpA* transcript for qRT-PCR analysis | - |
| *hrpA*_qRTPCR_rv | TACGGCCAGAGACCTCAA | reverse primer to amplify the *hrpA* transcript for qRT-PCR analysis | - |
| *fis*_qRTPCR_fw | TGCGTGACTCGGTTAAAC | forward primer to amplify the *fis* transcript for qRT-PCR analysis | - |
| *fis*_qRTPCR_rv | TTTCTTACGCAGCGTACC | reverse primer to amplify the *fis* transcript for qRT-PCR analysis | - |
| *lcrF*_qRTPCR_fw | GATTTCATGGCAGAGCAGC | forward primer to amplify the *lcrF* transcript for qRT-PCR analysis | - |
| *lcrF*_qRTPCR_rv | TTGGGTGTGGAGTTGTCTC | reverse primer to amplify the *lcrF* transcript for qRT-PCR analysis | - |
| *flhD*_qRTPCR_fw | CATGGCGGATGCATTATC | forward primer to amplify the *flhD*  transcript for qRT-PCR analysis | - |
| *flhD*_qRTPCR_rv | AGTGACTCGACAAGAGA | reverse primer to amplify the *flhD*  transcript for qRT-PCR analysis | - |
| *flhC*_qRTPCR_fw | TGCTCGTCTACAGATGCTTG | forward primer to amplify the *flhC*  transcript for qRT-PCR analysis | - |
| *flhC*_qRTPCR_rv | AACACCCGTGCAATAGCC | reverse primer to amplify the *flhC*  transcript for qRT-PCR analysis | - |
| *fliF*_qRTPCR_fw | ATTGTCGCCTTGATGCTC | forward primer to amplify the *fliF* transcript for qRT-PCR analysis | - |
| *fliF*_qRTPCR_rv | CAGCCGCAAGCGAGTTTCAT | reverse primer to amplify the *fliF* transcript for qRT-PCR analysis | - |
| *cheY*_qRTPCR_fw | AGAAGCCGAAGATGGTGT | forward primer to amplify the *cheY* transcript for qRT-PCR analysis | - |
| *cheY*_qRTPCR_rv | TGCGGTCACCATCAGAAC | reverse primer to amplify the *cheY* transcript for qRT-PCR analysis | - |
| *fis*_probe_fw | AACTCACAAGATCAGGTGAC | forward primer to amplify *fis* used for the synthesis of a *fis* probe | - |
| *fis*_probe_rv | GAAATTAATACGACTCACTATAGGGACCATGTCCAACAATGGCTG | reverse primer to amplify *fis* used for the synthesis of a *fis* probe | - |
| *yscM*_qPCR_fw | AATTACCCAAGTGGGACACG | forward primer to amplify the *yscM*  transcript for qPCR analysis | - |
| *yscM*_qPCR_rv | TCACTATCACTTCCCCTGCCT | reverse primer to amplify the *yscM*  transcript for qPCR analysis | - |
| *repA*_qPCR_fw | TATCAAACTGAACGTAAGGCTC | forward primer to amplify the *repA*  transcript for qPCR analysis | - |
| *repA*_qPCR_rv | AACGATAGCTTCAATGTCACG | reverse primer to amplify the *repA*  transcript for qPCR analysis | - |
| *glnA*_qPCR_fw | GGCATGGATCCTGAGATGA | forward primer to amplify the *glnA* transcript for qPCR analysis | - |
| *glnA*_qPCR_rv | CGTCAAAACATGTTCAGCGGAC | reverse primer to amplify the *glnA* transcript for qPCR analysis | - |
| *rpoB*_qPCR_fw | AATCGTCAAGGTGTATCTGG | forward primer to amplify the *rpoB* transcript for qPCR analysis | - |
| *rpoB*_qPCR_rv | TCGTAAGGCATATCTTCGAT | reverse primer to amplify the *rpoB* transcript for qPCR analysis | - |
| *YPK*_3178_qPCR_fw | ATCCACTGTTACCAGAATGC | forward primer to amplify the *YPK*_3178 transcript for qPCR analysis | - |
| *YPK*_3178_qPCR_rv | AGAAGAAACAACGGTCAAAA | reverse primer to amplify the *YPK*_3178 transcript for qPCR analysis | - |
| *yopH_int_fw* | TTTTGCGTGAAGGACTGCGT | forward primer to check pYV-cured strains | - |
| *yopH_int_rv* | TTTACTGCCATAGGTACCACTC | reverse primer to to check pYV-cured strains | - |
