# Supplementary material for: Fis suppresses late-stage virulence gene expression in Yersinia pseudotuberculosis at environmental temperatures
Source: PLoS Pathog. 2026 Mar 25;22(3):e1014105. doi: 10.1371/journal.ppat.1014105 (PMC13046262; doi:10.1371/journal.ppat.1014105)
Supplement: S3 Table — (DOCX) [file ppat.1014105.s003.docx]

**S3 Table. Temperature-dependent expression of motility-associated genes**. Average RNA-seq read counts for motility-associated genes in *Y. pseudotuberculosis* wild type grown at 25 and 37 °C are shown, along with the calculated read ratio of 25°/37°C.

| **gene** | **gene name** | **wt_e25°C_avarage reads**  **(RNA-seq)** | **wt_e37°C_avarage reads**  **(RNA-seq)** | **ratio 25°/37°C** |
| --- | --- | --- | --- | --- |
| YPK_1745 | ***flhD*** | 746.0 | 591.9 | **1.26** |
| YPK_1746 | ***flhC*** | 1151.1 | 650.7 | **1.77** |
| YPK_2378 | ***fliZ*** | 912.2 | 28.7 | **31.78** |
| YPK_2379 |  | 273.4 | 3.6 | **76.28** |
| YPK_2380 | ***fliA*** | 2577.1 | 72.8 | **35.42** |
| YPK_2381 | ***fliC*** | 10157.8 | 150.5 | **67.50** |
| YPK_2382 | ***fliD*** | 2684.4 | 35.7 | **75.24** |
| YPK_2383 | ***fliS*** | 404.2 | 7.4 | **54.70** |
| YPK_2384 | ***fliT*** | 303.5 | 5.6 | **53.89** |
| YPK_2390 | ***fliE*** | 1005.0 | 66.4 | **15.13** |
| YPK_2391 | ***fliF*** | 2661.6 | 60.8 | **43.80** |
| YPK_2392 | ***fliG*** | 1781.5 | 40.5 | **43.94** |
| YPK_2393 | ***fliH*** | 1013.6 | 17.1 | **59.38** |
| YPK_2394 | ***fliI*** | 1580.6 | 37.7 | **41.95** |
| YPK_2395 | ***fliJ*** | 494.3 | 11.8 | **41.91** |
| YPK_2396 | ***fliK*** | 1350.4 | 99.8 | **13.53** |
| YPK_2397 |  | 67.8 | 1.1 | **63.83** |
| YPK_2398 | ***fliL*** | 854.2 | 25.0 | **34.12** |
| YPK_2399 | ***fliM*** | 2555.2 | 54.2 | **47.15** |
| YPK_2400 | ***fliN*** | 239.5 | 3.8 | **62.82** |
| YPK_2401 | ***fliO*** | 640.3 | 19.4 | **33.08** |
| YPK_2402 | ***fliP*** | 693.7 | 24.1 | **28.74** |
| YPK_2403 | ***fliQ*** | 204.8 | 9.6 | **21.42** |
| YPK_2404 | ***fliR*** | 180.2 | 19.2 | **9.37** |
| YPK_2415 | ***flgL*** | 1827.1 | 114.5 | **15.96** |
| YPK_2416 | ***flgK*** | 2766.0 | 81.1 | **34.09** |
| YPK_2417 | ***flgJ*** | 1510.8 | 58.8 | **25.69** |
| YPK_2418 | ***flgI*** | 1224.2 | 29.7 | **41.28** |
| YPK_2419 | ***flgH*** | 1093.1 | 14.5 | **75.49** |
| YPK_2420 | ***flgG*** | 2234.3 | 31.9 | **69.94** |
| YPK_2421 | ***flgF*** | 1863.3 | 18.9 | **98.71** |
| YPK_2422 | ***flgE*** | 4262.0 | 79.2 | **53.80** |
| YPK_2423 | ***flgD*** | 2242.3 | 31.4 | **71.34** |
| YPK_2424 | ***flgC*** | 1174.8 | 16.9 | **69.45** |
| YPK_2425 | ***flgB*** | 1376.2 | 21.1 | **65.16** |
| YPK_2426 | ***flgA*** | 697.5 | 66.2 | **10.54** |
| YPK_2427 | ***flgM*** | 1012.6 | 115.8 | **8.75** |
| YPK_2428 | ***flgN*** | 982.6 | 112.8 | **8.71** |
| YPK_2429 | ***inv*** | 1516.1 | 209.6 | **7.23** |
| YPK_2430 | ***flhE*** | 190.2 | 6.9 | **27.42** |
| YPK_2431 | ***flhA*** | 1219.1 | 76.4 | **15.95** |
