# Supplementary figures and images for: Fis suppresses late-stage virulence gene expression in Yersinia pseudotuberculosis at environmental temperatures
Source: PLoS Pathog. 2026 Mar 25;22(3):e1014105. doi: 10.1371/journal.ppat.1014105 (PMC13046262; doi:10.1371/journal.ppat.1014105)

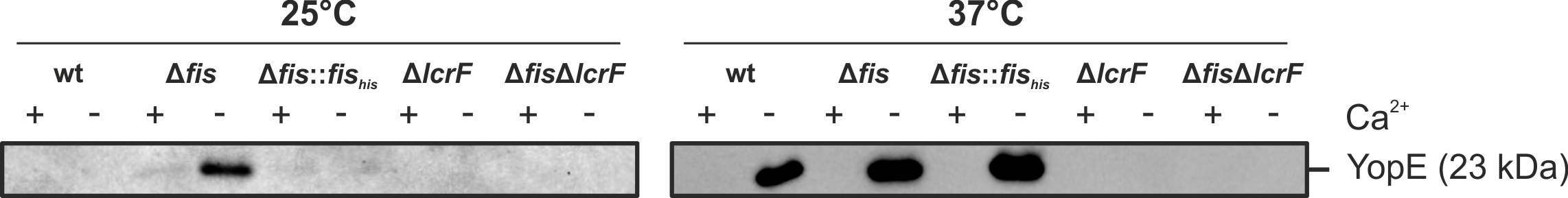

Supplement: S6 Fig — (TIF) [file ppat.1014105.s011.tif]
